# Manganese Stress Adaptation Mechanisms of *Bacillus safensis* Strain ST7 From Mine Soil

**DOI:** 10.3389/fmicb.2021.758889

**Published:** 2021-11-25

**Authors:** Xueqin Ran, Zhongmei Zhu, Hong Long, Qun Tian, Longjiang You, Xingdiao Wu, Qin Liu, Shihui Huang, Sheng Li, Xi Niu, Jiafu Wang

**Affiliations:** Key Laboratory of Plant Resource Conservation and Germplasm Innovation in Mountainous Region (Ministry of Education), College of Animal Science/Institute of Agro-Bioengineering, Guizhou University, Guiyang, China

**Keywords:** *Bacillus safensis*, manganese oxidation, transcriptome, soil, adaptation

## Abstract

The mechanism of bacterial adaption to manganese-polluted environments was explored using 50 manganese-tolerant strains of bacteria isolated from soil of the largest manganese mine in China. Efficiency of manganese removal by the isolated strains was investigated using atomic absorption spectrophotometry. *Bacillus safensis* strain ST7 was the most effective manganese-oxidizing bacteria among the tested isolates, achieving up to 82% removal at a Mn(II) concentration of 2,200 mg/L. Bacteria-mediated manganese oxide precipitates and high motility were observed, and the growth of strain ST7 was inhibited while its biofilm formation was promoted by the presence of Mn(II). In addition, strain ST7 could grow in the presence of high concentrations of Al(III), Cr(VI), and Fe(III). Genome-wide analysis of the gene expression profile of strain ST7 using the RNA-seq method revealed that 2,580 genes were differently expressed under Mn(II) exposure, and there were more downregulated genes (*n* = 2,021) than upregulated genes (*n* = 559) induced by Mn stress. KAAS analysis indicated that these differently expressed genes were mainly enriched in material metabolisms, cellular processes, organism systems, and genetic and environmental information processing pathways. A total of twenty-six genes from the transcriptome of strain ST7 were involved in lignocellulosic degradation. Furthermore, after 15 genes were knocked out by homologous recombination technology, it was observed that the transporters, multicopper oxidase, and proteins involved in sporulation and flagellogenesis contributed to the removal of Mn(II) in strain ST7. In summary, *B. safensis* ST7 adapted to Mn exposure by changing its metabolism, upregulating cation transporters, inhibiting sporulation and flagellogenesis, and activating an alternative stress-related sigB pathway. This bacterial strain could potentially be used to restore soil polluted by multiple heavy metals and is a candidate to support the consolidated bioprocessing community.

## Introduction

Manganese (Mn), one of the most abundant metals in the Earth’s crust, exerts a significant impact on the biogeochemical cycles of other metals, sulfur, and carbon and is considered the strongest naturally occurring oxidizing agent in the environment (Tebo et al., 2005). Furthermore, Mn is an essential trace element for all living organisms, acting as a cofactor for many enzymes, such as oxidoreductases, transferases, ligases, and hydrolases (Wang et al., 2019). Mn also participates in oxidative stress resistance, metabolism and decomposition of reactive oxygen species (ROS) (Bosma et al., 2021), and is required for the development and function of nerve and immune cells, control of blood sugar and vitamin levels in animals, and photosynthesis and respiration processes in plants (Gao et al., 2014). However, consumption of food or drinking water containing a high level of Mn has undesirable effects on human health (O’Neal and Zheng, 2015). Excessive Mn can accumulate in the human pancreas, bone, kidney, liver, adrenal, and pituitary glands, with toxic effects and a half-life of nine years in bones (O’Neal and Zheng, 2015). Mn toxicity is associated with dopaminergic dysfunction, and Parkinson’s and Alzheimer’s diseases (Bjørklund et al., 2019).

Excessive Mn in animals and plants predominantly results from a high level of Mn in the soil environment. There are seven valence states of Mn, and the dominant states in natural environments are Mn(II), Mn(III), and Mn(IV) oxides. Mn(II) is the main form toxic to life, and because it is soluble in water so it can accumulate in human cells via food chains. Mn in other valence states in natural environments can be transformed into Mn(II), which can easily permeate into ground water and contaminate the aquatic environment. Many countries, including Australia, Bangladesh, China, and the United States, have reported that the Mn concentration in ground water exceeds the permitted limit (Ali et al., 2017; McMahon et al., 2019; Adeyeye et al., 2020; Ghosh et al., 2020).

Mining and smelting are the two main source of contamination of ground water, rivers, streams, and lakes. In China, some of the largest Mn industries are built in Songtao county, northeast of Guizhou. The average amount of Mn(II) in the surface water of the Songtao Manganese Mine is 39.67-times higher than the safety standard for drinking water in China (0.1 mg/L), and almost half of the water sources in this area fail to meet the drinking water standard. Moreover, Mn pollution has impacted the growth and development of children living nearby. For remediation, chemical methods such as filtering with manganese sand are often used to reduce the high concentrations of Mn in wastewater from mine factories. However, not all Mn(II) is oxidized into Mn(III) or Mn(IV) by oxygen in chemical oxidation processes, and it is impossible for Mn metal to be completely eliminated via abiotic sedimentation and aeration (Li et al., 2019). Abiotic Mn(II) oxidation is thermodynamically favored at a higher pH, but many bacteria are capable of transforming soluble Mn(II) to its oxide precipitates of Mn(IV) oxides in neutral or acidic pH conditions (Bohu et al., 2015; Piazza et al., 2019). Manganese-oxidizing bacteria (MOB) harbor multicopper oxidase (MCO)-type enzymes, manganese oxidase, or heme peroxidase to catalyze Mn(II) oxidization (Anderson et al., 2009; Su et al., 2013; Das et al., 2020; Tang et al., 2021). The Mn(II) removal efficiency by enzymes of MOB is approximately five-times higher than that of oxidation reaction by O_2_ in natural environments (Katsoyiannis and Zouboulis, 2004; Su et al., 2013). Furthermore, some MOBs can survive in media with a Mn(II) concentration as high as 3,000 mg/L (Wan et al., 2017). In contrast, monkeys displayed abnormal cognition, motor functioning, and behavior when the Mn(II) concentration was only 29.4–73.7 μg/L in blood (Schneider et al., 2006). However, it remains to be elucidated how MOBs adapt to environments featuring a high concentration of toxic Mn. In the present study, MOBs were isolated from the soil of a manganese mine, and the response mechanism of these bacterial strains to manganese stress were explored.

## Materials and Methods

### Isolation and Screening of Manganese-Tolerant Bacterial Strains

Samples were collected from 0- to 20-cm depths of the subsoil located in the Songtao manganese mine (108°51′50″ E, 27°58′30″ N), Guizhou province, China. Soil in this area is heavily polluted with manganese as a result of anthropogenic mining activities; the total Mn in the sampled soils was in the range of 2,000–2,300 mg/kg. Soil samples were suspended in sterile water at a ratio of 1:9, and the mixtures were vigorously vortexed for 10 min. Approximately 80 μL supernatant was plated on PYCM solid medium [peptone 0.8 g/L, yeast extract 0.2 g/L, K_2_HPO_4_ 0.1 g/L, MgSO_4_⋅7H_2_O 0.2 g/L, NaNO_3_ 0.2 g/L, CaCl_2_ 0.1 g/L, (NH_4_)_2_CO_3_ 0.1 g/L, 1.5% agar, pH 7.0] (Tian et al., 2017) containing 200 mg/L MnCl_2_, and plates were incubated at 28°C for 7 days. Single colonies were selected and screened by plating on solid PYCM media containing MnCl_2_ concentrations from 700 mg/L up to 2,200 mg/L.

### Identification of Isolated Strains

Total genomic DNA was extracted from each isolated strain using a TIANamp Bacteria DNA Kit [Tiangen Biotech (Beijing) Co., China] according to the manufacturer’s protocol. The 16S rRNA and *gyrA* genes were amplified by PCR using the bacterial genomic DNA as templates (Weisburg et al., 1991). Purified PCR products were inserted into the pMD19-T vector (Takara Co., Japan), transformed into *Escherichia coli* DH5α and sequenced by the Sanger method. Consensus phylogenetic trees were reconstructed by the UPGMA method using the MEGA v7 program (Kumar et al., 2016b) with 1,000 bootstrap repetitions.

### Measurement of Manganese Removal Efficiency of Bacteria

Bacterial suspensions in exponential growth phase were inoculated in a 1:20 ratio into PYCM broth containing 250 mg/L MnCl_2_ and were cultured at 180 rpm and 28°C for 7 days. The culture supermatant from day 1 to day 7 was sampled and the Mn(II) in the samples was quantitated in triplicate using inductively coupled plasma-optical emission spectrometry (ICP-OES) (PQ9000, Analytik Jena, Germany). Mn removal efficiency was calculated via a previously reported equation (Shu et al., 2019).

### Growth of Bacterial Strain Under Mn(II) Stress

The isolated bacterial strain was cultured in PYCM liquid medium containing MnCl_2_ at concentrations of 0, 250, 500, 1,000, 1,500, and 2,200 mg/L. The optical density at 600 nm (OD_600_) and pH values of the bacterial suspension were recorded at various time points from 0 to 64 h. The biofilm formation of the strain was detected according to a previous description (Auger et al., 2006).

### Growth of Bacterial Strain in Media Containing Heavy Metals

The growth and tolerance of the selected bacterial strain to five heavy metals were measured according to the method reported by Oladipo et al. (2018). Briefly, stock solutions of heavy metal salts were prepared, including CdSO_4_, Al_2_(SO_4_), CuSO_4_, FeCl_3_, and K_2_Cr_2_O_7_. The bacterial strain was incubated at 180 rpm and 28°C for 72 h in PYCM liquid medium containing the different metal solutions at concentrations of 0, 100, 250, 500, and 1,000 mg/L. All tests were repeated four-times. The OD_600_ of the bacterial cultures was determined at 4, 8, 12, 16, 20, 24, 36, 48, and 72 h.

### Motility of Bacterial Strains Under Mn(II) Stress

A puncture was created in a plate of PYCM solid media, and the isolated bacterial strain was inoculated to determine bacterial motility under Mn stress. The plate contained 0.3% agar to assess swimming motility, while 0.6% agar was used to test swarming motility. An equivalent number of bacteria in exponential growth phase were inoculated into PYCM soft-agar plates supplemented with or without 250 mg/L Mn(II) and were incubated for 8–32 h. The diameter of the spreading colony was measured, and swimming/swarming areas were calculated according to a previous report (Hariri et al., 2016). Motility detection in mutants with flagellar genes knocked out utilized the same method as that of wild strain ST7 without Mn supplied in the media. All assays were performed in quadruplicate.

### Mn(II) Oxidation Activity Assays

The isolated strains were spread on solid-agar PYCM plates and cultured at 28°C for 7 days. Mn oxide products, including Mn(III) and Mn(IV), on plates or in liquid cultures were monitored by using the colorimetric dye solution of leucoberbelin blue (LBB; 0.04%, w/v) (Krumbein and Altmann, 1973). Manganese oxidation activity of strains was quantified by employing the LBB method and using KMnO_4_ solution for the standard curve as previously described (Johnson and Tebo, 2008).

### Visualization of Mn(II) Oxides by Scanning Electron Microscopy

Bacteria cultured at 28°C for 7 days in PYCM media (refer to Section “Isolation and screening of manganese-tolerant bacterial strains”) with 2,200 mg/L MnCl_2_ were collected and washed in phosphate-buffered saline (PBS, pH 7.4). Bacterial pellets were prepared and observed using scanning electron microscopy (SEM) as previously reported (Bohu et al., 2015). Attachments on the bacterial surface were observed via a Hitachi S-3400N scanning electron microscope with 20,000 V acceleration voltage.

### Analysis of Transcriptome Profile of Bacterial Strain Under Mn(II) Stress

The isolated bacterial strain was incubated in PYCM liquid medium in the absence or presence of 250 mg/L MnCl_2_. Three samples were collected from each culture: samples L01–L06 were from mid-exponential growth phase cultures while samples L07–L12 were from the onset of stationary phase cultures. Samples L01–L03, obtained in the absence of Mn(II), were collected after culture for 8 h [colony-forming unit (cfu)/mL = 3.183 × 10^8^], while samples L04–L06 were obtained following culture in the presence of Mn(II) for 16 h (cfu/mL = 3.182 × 10^8^). Samples L07–L09 were collected from media without Mn(II) after culture for 16 h (cfu/mL = 4.635 × 10^8^), while samples L10–L12 were obtained following culture in the presence of Mn(II) for 24 h (cfu/mL = 4.573 × 10^8^). Based on the protocol of Genedenovo Biotechnology Co., Ltd. (Guangzhou, China), 12 cDNA libraries were constructed and sequenced via an Illumina HiSeq^TM^ 2500 platform.

The raw data were filtered using the fastp Toolkit^1^ to remove low-quality sequences, which were sequences with > 10% unidentified nucleosides (N), sequences with > 50% bases containing a phred quality score < 20, or sequences containing only the barcode adapter. The remaining sequences were then aligned with the reference genome of *Bacillus safensis* KCTC 12796BP (assembly no. GCF_001895885.1, containing 4,058 genes) using the STAR v2.7 program^2^ and allowing no mismatches, and the sequences mapping to rRNA were removed. Expression patterns of genes were calculated according to the value of counts per million (CPM) to standardize the gene expression levels. The DESeq2 and edgeR packages in the R v4.0.4 platform^3^ were used to identify differentially expressed genes (DEGs) across groups. Taking the FDR (false discovery rate) value of 0.05 as the threshold, up- or downregulated genes were recognized if the value of |log2 (fold change)| was ≥ 1. These data were further displayed in a volcano plot created with the SangerBox program^4^ taking the FDR value and log2FC as coordinate axes. Nucleotide and amino acid sequences of the DEGs were generated from the assembled transcripts by Trinity v2.85 (Haas et al., 2013). Amino acid sequences of the DEGs were inputted for Kyoto Encyclopedia of Genes and Genomes (KEGG) orthology via the KEGG Automatic Annotation Server (KAAS)^5^. Gene lists from 30 species of the genus *Bacillus* containing 148,323 complete genome sequences were used as references. The expressed genes were further examined to find those related with lignocellulose degradation based on KAAS hierarchical categories and previously archived literature.

### Validation of Differentially Expressed Genes

The same aliquot of total RNA used for RNA-seq was also used to validate the DEGs by RT-qPCR. Gene-specific primers were designed using Primer 5.0 software (Supplementary Table S1) and using the 16S rRNA gene as the internal reference gene. The qPCR reaction was performed according to a previous study (Livak and Schmittgen, 2001). The corresponding RT-qPCR efficiency (E) was qualified in the range of 90.1–101.9% (Supplementary Figure S1). All assays were performed in triplicate.

### Gene Knockout by Homologous Recombination Technology

Homologous recombination technology was used to knock out 15 genes by single-cross integration as previously reported (Liu et al., 2019). In brief, the partial fragment F1 without the stop codon of the target gene was amplified from genomic DNA of wild-type *B. safensis* ST7. The complete kanamycin gene (fragment F2) was amplified from plasmid pPIC9K (Invitrogen Life Technologies) by PCR using primers km-F and km-R (Supplementary Table S1). The combined fragment F3, comprising the incomplete target gene (fragment F1) fused upstream of the kanamycin gene (fragment F2), was generated by PCR (Liu et al., 2019). After digestion with *Bam*HI, the F3 fragment formed a circular DNA and was electroporated into wild-type *B. safensis* ST7 cells.

The gene-knockout colonies were selected on solid PYCM media plates supplemented with 5 μg/mL kanamycin. The deletion of the target gene was verified by PCR tests and sequencing. The Mn(II) oxidation ability of each mutant (Δgene) was assessed by Mn(II) oxidation activity assays using the LBB and ICP-OES methods, as previously described (refer to Section “Mn(II) oxidation activity assays”), with the wild-type strain as a control. Mutants harboring knocked out flagellar genes were further used to detect motility, as described above (refer to Section “Motility of bacterial strains under Mn(II) stress”). Spore production was assayed in mutants containing deletions of sporulation-related genes according to the protocol of a spore staining kit (Shanghai Solarbio Bioscience & Technology Co., China, Cat no. G1132), using malachite green as the dye.

## Results

### Isolation and Identification of Manganese-Oxidizing Bacteria Strains

Soil was sampled from an abandoned manganese mine located in Songtao county, Guizhou, China, that had not performed smelting for 7 years, and 50 bacterial strains were isolated on media supplemented with 200 mg/L Mn(II) (Supplementary Table S2). The isolates were further tested in broth media containing different concentrations of Mn(II), and it was determined that seven strains were able to tolerate exposure to 1,800–2,200 mg/L Mn(II). These seven strains were characterized as *Rhizobium* sp., *Arthrobacter oxydans*, and *B. safensis* based on nucleotide sequence similarities of the 16S rRNA gene and gyrase subunit A gene (*gyrA*) (Supplementary Figures S2, S3).

### Determination of Manganese Absorption and Oxidation Ability of the Isolated Strains

The isolated bacterial strains were investigated for their manganese removal efficiencies in PYCM media. After incubation for 7 days, the bacterial isolates presented manganese removal efficiencies ranging from 11 to 82% (Supplementary Table S2), with strain ST7 displaying the highest Mn removal rate. Higher efficiencies of manganese absorption were detected from day 3 until day 7 (Supplementary Figure S4). The Mn(II) oxides generated by isolate ST7 were further measured by LBB solution based on the characteristics of LBB dye being specifically oxidized by Mn(III) and Mn(IV) (Krumbein and Altmann, 1973).

A blue color appeared in an ST7 colony cultured on solid media supplemented with 250 mg/L MnCl_2_, and the color became much deeper on the plate containing 2,200 mg/L MnCl_2_ (Supplementary Figure S5). The oxidation capacity of isolate ST7 was high, with a manganese oxide biomass of 7.75 μM/d. Numerous irregular precipitates covering the surface of isolate ST7 were visible by SEM after incubation for 7 days in 2,200 mg/L MnCl_2_ liquid media (Supplementary Figure S5). Strain ST7 was thus characterized as a MOB with a high capacity for Mn removal. Furthermore, the swimming motility of strain ST7 was stimulated by Mn(II) in a 0.3% soft-agar plate supplemented with 250 mg/L Mn(II) (Supplementary Figure S6).

### Growth of Strain ST7 Under Mn(II) Stress

Strain ST7 grew rapidly in PYCM medium without manganese (Supplementary Figure S7), reaching mid-exponential phase in 8 h and stationary phase in 16 h. When the strain was cultured in media supplemented with 2,200 mg/L MnCl_2_, the time to stationary phase increased to 44 h. The growth rate of strain ST7 in 250 mg/L MnCl_2_ was reduced to half of that of the control. Thus, 250 mg/L MnCl_2_ was considered as the half-effective dosage (ED_50_) for strain ST7. Furthermore, in PYCM media with a small amount of peptone and yeast extracts as an energy source, an increase in the pH value of cultures was observed during the exponential growth phase of strain ST7, which later returned to the initial pH of 7 at the stationary growth phase for all three cultures with or without Mn(II) stress (Supplementary Figure S8). In addition, the biofilm formation of strain ST7 was obviously promoted by the presence of Mn(II). After culturing in LB media for 72 h in a microtiter plate, the OD_595_ value of strain ST7 under Mn(II) stress was 3.340 ± 0.699, and was 0.715 ± 0.036 in the control wells lacking Mn(II) (Supplementary Figure S9).

### Maximum Tolerance of Strain ST7 to Heavy Metals

The growth of strain ST7 was measured in minimal PYCM media containing heavy metals at various concentrations. Strain ST7 grew in media supplemented with Al(III), Cr(VI), or Fe(III), but not in media containing Cd(II) or Cu(II). The maximum metal tolerance of strain ST7 to Al(III), Cr(VI), and Fe(III) was 500 mg/L, 250 mg/L, and 250 mg/L, respectively (Supplementary Figure S10).

### Expression Profile of *Bacillus safensis* ST7 During Mn(II) Exposure

To investigate the response mechanism of strain ST7 to manganese stress, 12 cDNA libraries were constructed from samples cultured in the presence or absence of 250 mg/L Mn(II). Whole-transcriptome gene expression profiles were analyzed using an Illumina HiSeq X-ten platform. All cDNA libraries generated 159.1 × 10^6^ clean sequences after quality control and filtering. There were similar matching percentages for all samples, with an average of 80.77% of sequences mapping onto the reference genome of *B. safensis*, and the Q30 was greater than 89.34% (Table 1).

**TABLE 1:** Statistical statement of transcriptome sequencing of *B. safensis* strain ST7.

Samples	Clean data (Gbp)	Obtained reads	Mapped reads	Mapping ratio (%)	Q30 (%)	GC (%)
L01	3.66	12343385	10940366	88.6%	89.37	43.17
L02	5.29	17844906	15975336	89.5%	89.34	43.45
L03	4.57	14566987	13652398	87.7%	89.35	43.33
L04	2.88	9787928	8611058	88.0%	89.64	43.40
L05	4.37	14746281	13015444	88.3%	89.76	43.05
L06	3.59	11658945	10965238	88.1%	89.71	43.32
L07	3.98	13898878	9666959	70.0%	94.51	48.22
L08	3.78	13087400	10499441	80.2%	94.00	43.70
L09	3.85	13563148	10156874	77.1%	94.32	45.67
L10	3.61	12635044	8635796	68.3%	94.30	45.87
L11	3.55	12393004	8985159	72.5%	94.22	45.56
L12	3.57	12596487	8856214	70.9%	94.29	45.79

**FIGURE 1:**
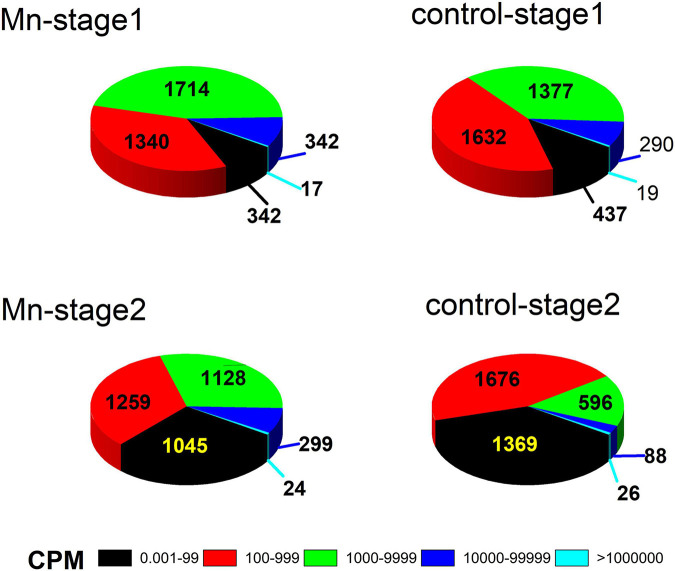
Gene expression patterns of strain ST7 in the presence of Mn(II) at exponential (stage 1) and stationary (stage 2) growth phases.

**FIGURE 2:**
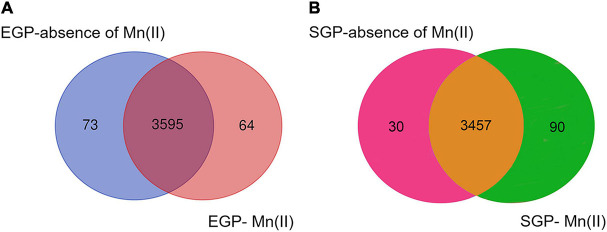
Venn diagram showing the overlap of numbers of expressed genes in strain ST7 in the presence or absence of 250 mg/L Mn(II). Numbers of expressed genes in **(A)** exponential growth phase (EGP) and **(B)** stationary growth phase (SGP) are indicated.

**FIGURE 3:**
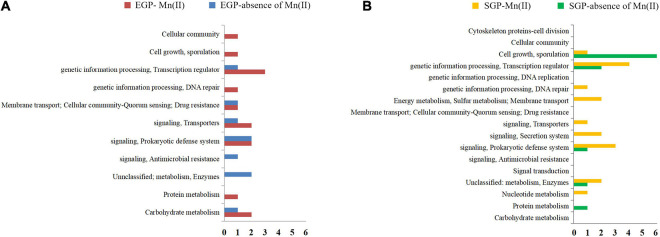
Enriched pathways of genes affected by manganese exposure. **(A)** Enriched pathways of genes expressed during the exponential growth phase (EGP) of strain ST7 in the presence or absence of Mn(II). **(B)** Enriched pathways of genes expressed during stationary growth phase (SGP) of strain ST7 in the presence or absence of Mn(II).

A total of 3,755 genes was annotated from the 12 libraries. The ratios of the expressed genes were 85.74–90.18% of the total reference genes (4,067). The normalized CPM data are listed in Supplementary Table S3. The expression levels for most genes were lower than 10,000 CPM (Figure 1). The expression profiles of strain ST7 differed in the absence and presence of Mn(II) (Figure 1). In total, 3,668 genes were expressed in the absence of Mn(II) at the mid-exponential growth phase. However, in the presence of Mn(II), the expression of 53 genes that functioned in the process of antimicrobial resistance was completely inhibited. Additionally, the expression of 64 genes enriched in the processes of transporting and DNA repair was induced by Mn(II) (Figures 2, 3). During the stationary growth phase, the numbers of expressed genes decreased to 3,487 and 3,547 in the absence and presence of Mn(II), respectively. There were 142 genes expressed in the presence of Mn(II) in mid-exponential growth phase that were not detected in the stationary growth phase. These included genes linked to the systems of membrane transport and secretion signaling processes.

**FIGURE 4:**
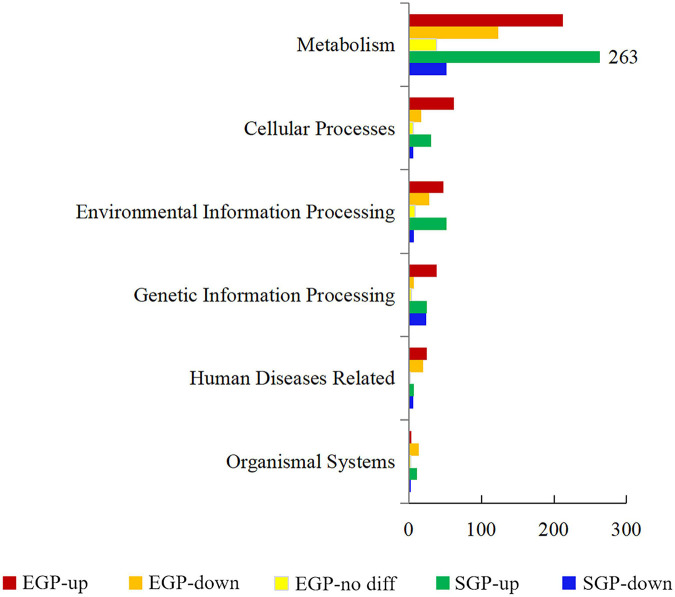
Enrichment hierarchies of differentially expressed genes (DEGs) in strain ST7 under manganese stress. The horizontal ordinate represents the number of enriched genes in each hierarchy. Numbers of enriched DEGs that were upregulated, downregulated, and without difference in exponential growth phase (EGP) are indicated as EGP-up (the upregulated DEGs at EGP), EGP-down (the downregulated DEGs at EGP), and EGP-no diff (genes with no significance at EGP), respectively. Numbers of DEGs that were upregulated and downregulated in stationary growth phase (SGP) are shown as SGP-up (the upregulated DEGs at SGP) and SGP-down (the downregulated DEGs at SGP), respectively.

Two software packages, edgeR and DESeq2, were used to analyze the DEGs. In total, 2,580 genes were differentially expressed in the presence and absence of Mn(II) (Supplementary Table S4). Based on KAAS analysis, the DEGs were enriched in six hierarchies including metabolisms, genetic information processing, environmental information processing, cellular processes, organism systems, and human disease-related processes (Figure 4). The enrichment categories of highly expressed DEGs with CPM above 1,000 are listed in Supplementary Table S5.

**FIGURE 5:**
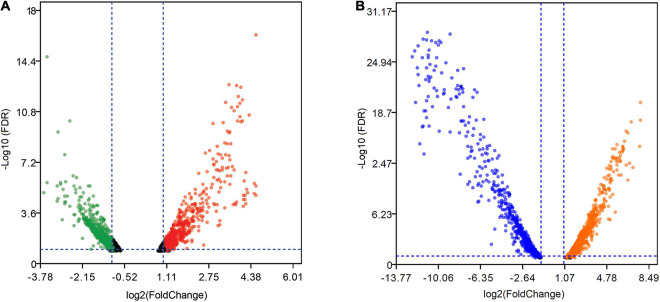
Volcano plots of DEGs in strain ST7 under manganese stress. **(A)** DEGs in mid-exponential growth phase. **(B)** DEGs in the onset of stationary growth phase. Green and blue dots represent the downregulated genes, while red and orange dots represent the upregulated genes.

At the mid-exponential growth phase, 1,205 DEGs were induced by Mn(II) and exhibited | logFC| ≥ 1, and of these genes, 599 were upregulated and 606 were downregulated (Figure 5A). The range of log2FC values varied from −3.82 to 5.99. The upregulated DEGs were enriched in pathways of energy metabolism, nucleotide metabolism, translation, and flagellar assembly (Supplementary Table S5), while the downregulated genes were clustered in quorum sensing, peptidoglycan biosynthesis, and propanoate metabolism. In the stationary growth phase (Supplementary Table S4), many more DEGs (2,133) with greater changes of log2FC values from −13.76 to 8.62 were identified compared with the DEGs from the mid-exponential growth phase. Of the DEGs in the stationary growth phase, 1,078 genes were upregulated and 1,055 genes were downregulated in the presence of Mn(II) (Figure 5B). Genes related to the metabolism of pyruvate and propanoate were upregulated, while those connected with ribosome function were largely inhibited by Mn(II) exposure (Supplementary Table S5).

The top 20 genes with the highest expression levels were analyzed further (Tables 2, 3). Of these, ten genes did not show significant difference in the absence or presence of Mn(II) at the exponential growth phase, and these genes were involved in processes such as carbohydrate metabolism, lipid metabolism, cellular community in quorum sensing, and transcription. However, the expression of four genes related to carbohydrate and lipid metabolisms, and cellular community in quorum sensing was significantly increased by Mn(II) stress, and six genes involved in sporulation and protein metabolism were significantly downregulated during Mn(II) exposure. When the ST7 growth reached its stationary phase, most of the top 20 genes were upregulated, including 23S rDNA and genes enriched in the processes of transcription, amino acid metabolism, carbohydrate metabolism, lipid metabolism, exosomes, and transporters. Only three genes of ncRNAs and transfer-messenger RNA were downregulated during the stationary period.

**TABLE 2:** Top 20 highly expressed genes in the mid-exponential growth phase of strain ST7.

No.	Gene-ID	Mean-control	Mean-Mn (II)	logFC	Up/down	Protein_ID	Product	Group from	KAAS path
1	RS10445	88280.22	141677.22	0.22	no-diff	WP_024422684.1	aldehyde dehydrogenase family protein	Mn (II) presence	Carbohydrate metabolism
2	RS11640	100730.83	176474.87	0.35	no-diff	WP_024422883.1	tetratricopeptide repeat protein	both	Cellular community – prokaryotes, Quorum sensing
3	RS00700	104162.21	130477.58	–0.16	no-diff	WP_024425592.1	DNA-directed RNA polymerase subunit beta′	both	Genetic Information Processing, Transcription
4	RS00695	91830.33	104047.58	–0.30	no-diff	WP_024425866.1	DNA-directed RNA polymerase subunit beta	Mn (II) presence	Genetic Information Processing, Transcription
5	RS16485	49900.61	92696.21	0.36	no-diff	WP_044331271.1	acyl-CoA dehydrogenase	Mn (II) presence	Lipid metabolism
6	RS14625	304383.81	293470.15	–0.51	no-diff	WP_073206977.1	fatty acid desaturase	both	Lipid metabolism, Lipid biosynthesis proteins
7	RS00720	42836.31	112770.20	0.89	no-diff	WP_024425594.1	elongation factor G	Mn (II) presence	genetic information processing, Translation factors
8	RS16905	709030.83	686287.16	–0.52	no-diff	tmRNA	transfer-messenger RNA	both	
9	RS00140	542029.69	658780.54	–0.19	no-diff	ncRNA	signal recognition particle sRNA large type	both	
10	RS04460	68343.95	97151.59	0.07	no-diff	WP_012009371.1	hypothetical protein	Mn (II) presence	
11	RS09610	8147.14	106043.44	3.23	up	WP_024425140.1	aconitate hydratase AcnA	Mn (II) presence	Carbohydrate metabolism
12	RS09515	22869.93	136468.06	2.12	up	WP_024425124.1	trypsin-like serine protease	Mn (II) presence	Cellular community – prokaryotes, Quorum sensing
13	RS16495	33687.41	97529.70	1.03	up	WP_044331273.1	3-hydroxyacyl-CoA dehydrogenase	Mn (II) presence	Lipid metabolism
14	RS03925	7328.75	172637.44	4.15	up	WP_024425256.1	hypothetical protein	Mn (II) presence	
15	RS14680	585070.82	249768.46	–1.69	down	WP_024424829.1	alpha/beta-type small acid-soluble spore protein	both	cellular processes, Cell growth, sporulation
16	RS11215	514610.92	158678.55	–2.15	down	WP_008344534.1	stage IV sporulation protein A	both	cellular processes, Cell growth, sporulation
17	RS04855	263511.07	118772.43	–1.59	down	WP_024425744.1	alpha/beta-type small acid-soluble spore protein	both	cellular processes, Cell growth, sporulation
18	RS13255	290406.13	102815.80	–1.96	down	WP_073206510.1	SafA/ExsA family spore coat assembly protein	both	cellular processes, Cell growth, sporulation
19	RS05125	372823.66	110835.29	–2.19	down	WP_044335827.1	S8 family serine peptidase	both	Protein metabolism
20	RS10900	300404.97	182576.48	–1.20	down	ncRNA	RNase P RNA component class B	both	
21	RS04210	159812.20	81786.51	–1.41	down	WP_012009334.1	gamma-type small acid-soluble spore protein	Mn (II) presence	cellular processes, Cell growth, sporulation
22	RS08325	133133.55	50844.61	–1.83	down	WP_024424105.1	outer spore coat protein CotE	Mn (II) presence	cellular processes, Cell growth, sporulation
23	RS13665	144372.34	50222.53	–1.99	down	WP_073206706.1	LysM peptidoglycan-binding domain-containing protein	Mn (II) presence	cellular processes, Cell growth, sporulation
24	RS18780	114554.98	49633.09	–1.66	down	WP_024426405.1	spore coat protein GerQ	Mn (II) presence	cellular processes, Cell growth, sporulation
25	RS04580	101499.85	43091.09	–1.69	down	WP_034623898.1	YhcN/YlaJ family sporulation lipoprotein	Mn (II) presence	cellular processes, Cell growth, sporulation
26	RS04525	225740.47	68288.06	–2.17	down	WP_024425326.1	PrkA family serine protein kinase	Mn (II) presence	signaling
27	RS12630	108308.45	28956.33	–2.35	down	WP_024423047.1	late competence protein ComER	Mn (II) presence	signaling and cellular processes, Secretion system
28	RS02490	110584.14	37469.98	–2.01	down	WP_087975235.1	outer membrane lipoprotein carrier protein LolA	Mn (II) presence	
29	RS04895	99559.03	28529.70	–2.26	down	WP_039177545.1	Cof-type HAD-IIB family hydrolase	Mn (II) presence	

**TABLE 3:** Top 20 highly expressed genes in the stationary growth phase of strain ST7.

No.	GENE-ID	Mean-control	Mean-Mn(II)	logFC	Up/down	Protein_ID	Product	Group from	KAAS path
1	RS00695	40759.24	154928.03	0.02	no-diff	WP_024425866.1	DNA-directed RNA polymerase subunit beta	Mn (II) presence	genetic information processing, Transcription
2	RS01065	20578.52	120265.77	0.65	no-diff	WP_024424449.1	RNA polymerase sigma factor SigW	Mn (II) presence	genetic information processing, Transcription
3	RS01825	3255.07	235980.93	4.31	up	WP_024424316.1	L-glutamate gamma-semialdehyde dehydrogenase	Mn (II) presence	Amino acid metabolism
4	RS17060	2429.32	116252.03	3.60	up	WP_034280960.1	ornithine–oxo-acid transaminase	Mn (II) presence	Amino acid metabolism
5	RS01090	4581.38	165966.12	3.53	up	WP_073203523.1	glutamine–fructose-6-phosphate transaminase (isomerizing)	Mn (II) presence	Carbohydrate metabolism
6	RS09610	17629.67	675703.72	3.42	up	WP_024425140.1	aconitate hydratase AcnA	Mn (II) presence	Carbohydrate metabolism
7	RS15120	60198.88	575277.27	1.26	up	WP_073207096.1	phosphoenolpyruvate carboxykinase (ATP)	Mn (II) presence	Carbohydrate metabolism, Citrate cycle (TCA cycle)
8	RS08280	15866.75	281071.94	2.69	up	WP_003211958.1	ribonuclease Y	Mn (II) presence	genetic information processing, Transcription
9	RS16485	33904.10	273456.59	1.66	up	WP_044331271.1	acyl-CoA dehydrogenase	Mn (II) presence	Lipid metabolism
10	RS10445	7859.07	226258.37	3.00	up	WP_024422684.1	aldehyde dehydrogenase family protein	Mn (II) presence	Lipid metabolism, Fatty acid degradation
11	RS14255	5833.30	122023.16	2.88	up	WP_041115046.1	AMP-binding protein	Mn (II) presence	Lipid metabolism, Lipid biosynthesis proteins
12	RS14625	16040.21	415515.37	2.83	up	WP_073206977.1	fatty acid desaturase	Mn (II) presence	Lipid metabolism, Lipid biosynthesis proteins
13	RS14235	10180.79	119964.21	2.04	up	WP_073206874.1	electron transfer flavoprotein subunit alpha/FixB family protein	Mn (II) presence	signaling and cellular processes, Exosome
14	RS02135	1463.53	129972.98	5.21	up	WP_073203824.1	copper-binding protein	Mn (II) presence	signaling, Transporters
15	RS12525	6510.92	125036.76	2.55	up	WP_024423028.1	GatB/YqeY domain-containing protein	Mn (II) presence	
16	RS00505	30765.76	578808.06	1.80	up	rRNA	23S ribosomal RNA	Mn (II) presence	
17	RS10400	3424.20	372107.78	5.09	up	WP_024422678.1	LysM peptidoglycan-binding domain-containing protein	Mn (II) presence	
18	RS03530	273378.40	274.25	–11.59	down	WP_024424576.1	glycosyltransferase family 2 protein	Mn (II) presence	cellular processes, Cell growth
19	RS05770	586115.35	793.10	–11.19	down	WP_003211658.1	spore coat protein	Mn (II) presence	cellular processes, Cell growth
20	RS05775	897215.34	1648.60	–11.00	down	WP_025093420.1	spore coat protein	Mn (II) presence	cellular processes, Cell growth
21	RS05785	241924.10	248.41	–11.23	down	WP_073204750.1	spore coat protein	Mn (II) presence	cellular processes, Cell growth
22	RS05800	494968.25	131.37	–13.82	down	WP_024426457.1	YjcZ family sporulation protein	Mn (II) presence	cellular processes, Cell growth
23	RS10620	370929.30	470.79	–11.28	down	WP_073205976.1	glycosyltransferase family 2 protein	Mn (II) presence	cellular processes, Cell growth
24	RS10630	734369.99	587.65	–12.03	down	WP_024422714.1	hypothetical protein	Mn (II) presence	cellular processes, Cell growth
25	RS10635	1476138.60	1393.91	–11.89	down	WP_073205979.1	glycosyltransferase	Mn (II) presence	cellular processes, Cell growth
26	RS12760	246513.96	336.13	–11.85	down	WP_073206394.1	hypothetical protein	Mn (II) presence	cellular processes, Cell growth
27	RS18765	181704.80	1129.61	–9.33	down	WP_073207818.1	spore coat protein	Mn (II) presence	cellular processes, Cell growth
28	RS08455	610095.51	934.65	–11.62	down	WP_024427042.1	N-acetylmuramoyl-L-alanine amidase	Mn (II) presence	Drug resistance: antimicrobial
29	RS06375	924349.33	14976.13	–8.29	down	WP_024424648.1	S8 family peptidase	Mn (II) presence	Protein metabolism
30	RS08370	472662.12	1063.17	–11.01	down	WP_073205255.1	S8 family peptidase	Mn (II) presence	Protein metabolism
31	RS05810	188067.82	856.92	–9.76	down	WP_073204753.1	ATP-dependent helicase	Mn (II) presence	Replication and repair
32	RS10900	909351.71	594020.50	–3.14	down	ncRNA	RNase P RNA component class B	both	
33	RS00140	1028660.45	140764.62	–5.50	down	ncRNA	signal recognition particle sRNA large type	both	
34	RS16905	1404852.99	953995.05	–3.13	down	tmRNA	transfer-messenger RNA	both	
35	RS03525	199854.22	153.97	–11.98	down	WP_073204217.1	hypothetical protein	Mn (II) presence	
36	RS03535	187801.06	289.50	–10.98	down	WP_044335009.1	hypothetical protein	Mn (II) presence	
37	RS03675	288211.08	842.10	–10.36	down	WP_167364607.1	collagen-like repeat preface domain-containing protein	Mn (II) presence	

It was interesting that 26 genes from the transcriptome of strain ST7 were predicted to be related to lignocellulosic degradation (Supplementary Table S6). Of these, genes coding for three main types of cellulases were expressed at a high level, especially in media without Mn(II) stress, which included endoglucanases, β-glucosidases, and cellobiohydrolase. The other 15 genes participated in cellobiose transportation, and production of ethanol, acetate, and lactate from cellulose. It was ascertained that approximately six genes were hemicellulases such as xylanase, according to the hierarchies of KAAS enrichment analysis. Furthermore, two genes coding for manganese catalase and multicopper oxidase domain-containing protein (MCO) might participate in lignin degradation, which was the case for manganese peroxidase and bacterial laccase based on a homology blast with *Bacillus pumilus* (Kumar et al., 2016a; Ogonda et al., 2021).

### Validation of RNA-Seq Data by RT-qPCR

**FIGURE 6:**
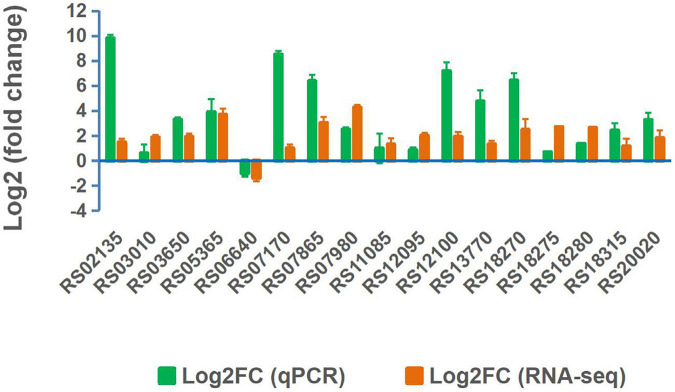
RT-qPCR validation of expressed genes in strain ST7 cultured in media supplemented with 250 mg/L Mn(II).

**FIGURE 7:**
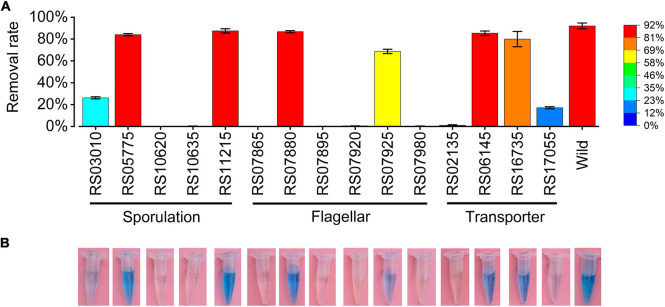
Manganese oxidation ability of mutants. Mutants with deleted genes and the wild-type strain were cultivated in PYCM liquid media supplemented with 250 mg/L Mn(II) for 7 days. **(A)** Manganese removal rates of mutants detected by ICP-OES method. **(B)** Manganese oxidation abilities of mutants detected by adding 150 μL of 0.04% LBB solution into the culture supernatants.

Seventeen expressed genes from the RNA-seq data were selected for validation by RT-qPCR. The expression patterns obtained by RT-qPCR for these genes were consistent with the trends of transcriptomic abundance changes based on the RNA-seq data (Figure 6). To demonstrate the function of the DEGs in Mn(II) oxidation, 15 DEGs with high CPM values or high differential fold-changes were knocked out from the wild-type ST7 genome using homologous recombination technology by a single exchange (Figure 7 and Table 4). First, sporulation and Mn(II) oxidation abilities were detected in mutants of five sporulation-related genes. Sporulation and Mn(II) oxidation were completely abolished in two mutants, Δ*RS10620* (glycosyltransferase family 2 protein) and Δ*RS10635* (glycosyltransferase) (Figure 7 and Supplementary Figure S11), while the Mn(II) oxidation ability decreased by 55.78% in mutant Δ*RS03010* (deletion of MCO-domain-containing protein). However, the sporulation and Mn(II) oxidation abilities were not affected by deletions of the *RS05775* or *RS11215* genes, which encode spore coat protein and stage IV sporulation protein A, respectively.

**TABLE 4:** Information of fifteen genes knocked out via homologous recombination technology.

				Exponential growth phase			Stationary phase						
No.	Gene_ID	Start-end	Strand	Mean (Mn (II) presence)	Mean (control)	logFC	Mean (Mn (II) presence)	Mean (control)	logFC	Protein_ID	Gene description	Homology %	Function enriched path
1	RS03010	598389– 599921	–	7927.584	2414.668	1.701	160.979	11144.304	–6.068	WP_0732 04075.1	multicopper oxidase domain-containing protein	86.42–99.61%	Sporulation
2	RS05775	1177165– 1177659	–				872.442	1734876.440	–10.916	WP_0250 93420.1	spore coat protein	86.77–99.19%	Sporulation
3	RS10620	2091926– 2093221	–	766.468	5935.665	–2.955	251.949	617644.697	–11.239	WP_0732 05976.1	glycosyltransferase family 2 protein	81.58–99.69%	Sporulation
4	RS10635	2094184– 2095146	+	6398.866	17954.557	–1.492	751.492	2751491.883	–11.804	WP_0732 05979.1	glycosyltransferase	80.39–99.58%	Sporulation
5	RS11215	2195294– 2196772	–	133193.056	596185.222	–2.159	85.830	9906.308	–6.815	WP_0083 44534.1	stage IV sporulation protein A	76.61–100%	Sporulation
6	RS07865	1572576– 1574168	+	12472.031	1493.585	3.066				WP_0732 05172.1	flagellar basal body M-ring protein FliF	71.15–100%	Flagella
7	RS07880	1575950– 1577266	+	7395.216	707.714	3.387	1841.286	4691.249	–1.344	WP_0732 05178.1	flagellar protein export ATPase FliI	88.84–99.54%	Flagella
8	RS07895	1578433– 1579758	+	7398.993	637.551	3.541				WP_0732 05181.1	flagellar hook-length control protein	84.29–99.30%	Flagella
9	RS07920	1581727– 1582725	+	8424.999	544.637	3.951	1905.595943	3859.086	–1.000	WP_0244 24167.1	flagellar motor switch protein FliM	76.72–99.90%	Flagella
10	RS07925	1582715– 1583836	+	7159.875	433.388	4.0495	1490.866	4433.245	–1.529	WP_0244 24166.1	flagellar motor switch phosphatase FliY	90.76–99.64%	Flagella
11	RS07980	1592875– 1594902	+	7623.183	392.277	4.276	1874.407	5558.474	–1.549	WP_0244 24158.1	chemotaxis protein CheA	90.05–99.15%	Flagella
12	RS02135	446150– 447784	–	39479.819	13707.704	1.530	67335.879	1925.328	5.118	WP_0732 03824.1	copper-binding protein	85.88–99.38%	Transporter
13	RS06145	1239207– 1240580	+				20440.359	687.676	4.898	WP_0732 04865.1	PTS galactitol transporter subunit IIC	89.37–99.93%	Transporter
14	RS16735	3223044– 3224096	–	4345.889	1371.874	1.654	41210.218	615.979	6.098	WP_0244 23451.1	iron ABC transporter permease	86.44–99.90%	Transporter
15	RS17055	3281661– 3283070	–				33453.736	705.125	5.590	WP_0250 94035.1	amino acid permease	87.24–100%	Transporter

Next, manganese oxidation activity and motility were detected in the mutants of six flagellar genes (Supplementary Figure S12). The swimming abilities of mutants Δ*RS07925*, Δ*RS07865*, and Δ*RS07880* decreased by 87.13, 81.83, and 68.78% respectively, demonstrating that these flagellar genes were crucial for the motility of isolate ST7. Four mutants (Δ*RS07865*, Δ*RS07895*, Δ*RS07920*, and Δ*RS07980*) lost their Mn(II) oxidation abilities. These genes encode the flagellar basal body M-ring protein, flagellar hook-length control protein, flagellar motor switch protein, and the chemotaxis protein, respectively. No obvious changes were detected in the Mn(II) oxidation abilities of the other two mutants, Δ*RS07880* and Δ*RS07925*.

Finally, Mn(II) oxidation abilities were detected in mutants of four transporter genes. Mn(II) oxidation was completely abolished in mutant Δ*RS02135*, while mutant Δ*RS17055* exhibited decreased efficiency in Mn(II) oxidation from 82% to 17.11%. Disruption of the other two genes, *RS06145* and *RS16735*, encoding phosphotransferase system (PTS) galactitol transporter subunit IIC and iron ATP-binding cassette (ABC) transporter permease, respectively, did not have any effect on Mn(II) oxidation.

## Discussion

### Isolation of Manganese-Oxidizing Bacteria From Soil

Toxic Mn(II) can be removed from different aqueous environments by MOB such as *Bacillus* sp. strain SG-1 (de Vrind et al., 1986), *Leptothrix discophora* strain SS-1 (Corstjens et al., 1997), *Flavobacterium* spp. (Hou et al., 2020), *Exiguobacterium* sp. CNU020 (Lee et al., 2009), and *Pseudomonas putida* strains MnB1 and GB-1 (Okazaki et al., 1997). In addition, several MOB—namely, *Pseudomonas* sp. nov., *Rhizobium* spp. (Moy et al., 2003), *Bacillus cereus* CP133 (Wang et al., 2016), *Bacillus megaterium* 1Y31 (Zhang et al., 2015), *Acinetobacter* spp. (Ren et al., 2019), and *Citrobacter freundi* (Tang et al., 2014)—were isolated from alfisol soil or mine soil. In the current study, 50 manganese-tolerant bacterial strains were isolated from the soil of the Songtao manganese mine (Supplementary Table S2), the largest mine in Asia. Most of the isolated strains were bacteria that are commonly distributed in soil, and the dominant genus was *Bacillus*. Some of the isolated bacterial taxa had previously been recognized for their abilities and characteristics related to manganese oxidation; the exceptions were *Arthrobacter oxydans* and *B. safensis*. The isolated bacteria showed diversity in Mn(II) removal efficiency even if the isolates were classified in the same genus.

Of the strains isolated in the current study, *B. safensis* strain ST7 was the most effective MOB. The maximum Mn(II) concentration tolerated by strain ST7 was 2,200 mg/L, and thus, up to 82% removal of Mn(II) was achieved, with production of 7.75 μM/d biomass of manganese oxides [Mn(III)/Mn(IV)]. The Mn(II) tolerance ability of strain ST7 was close to that of *Arthrobacter* sp. strain HW-16 (oxidation ratio of 66.28% in 3,000 mg/L Mn(II) media) (Wan et al., 2017), and much higher than that of *B. cereus* strain P1 (Fan et al., 2016) and *Streptomyces spinoverrucosus* strain NB-7 (Wang et al., 2013).

The current study demonstrated that strain ST7 can also tolerate multi-metal stress. The manganese mine region in Songtao county is polluted with numerous heavy metals, such as Cd(II), Al(III), Cr(VI), Cu(II), Fe(III). Therefore, the growth of *B. safensis* strain ST7 was explored in media containing these heavy metals. Isolate ST7 grew in the presence of three of these heavy metals, tolerating Al(III) at 500 mg/L, and Cr(VI) and Fe(III) at 250 mg/L. The maximum metal tolerance of strain ST7 was much higher than the threshold records in soil (Kabata-Pendias, 2010), as it existed in sandy soil with 1,000–2,223 μg/L iron at pH 2.5–4.5, and in soil with 5,700 μg/L Al at pH 4.4. The background content of Cr in soil ranged from 14 to 109 mg/kg, and the trigger action value (TAV) for Cr(VI) is 3–25 mg/kg. Moreover, the tolerance capacity of strain ST7 was higher than that of *B. cereus* and *Bacillus kochii* for Al(III) and Fe(III) from mining sites (Oladipo et al., 2018), and higher than that of *Bacillus* sp. S3 for Cr(VI) from soil (Zeng et al., 2020). Hexavalent chromium [Cr(VI)] is a strong oxidant, teratogen, and carcinogen that easily crosses biomembranes and is toxic to the proteins, genomic DNA, and lipids of bacteria (Pushkar et al., 2021). The solubility of aluminum is increased in acidic soil, and Al(III) toxicity severely reduced plant growth and productivity globally (Gu et al., 2021). Strain ST7 might therefore be considered as a valuable resource for bioremediation of multiple heavy metals.

### Gene Expression Profiles of Strain ST7 Under Mn(II) Stress

In a previous study (Hoover et al., 2010), it was proposed that the different capabilities for Mn removal might be related to diverse genetic mechanisms and patterns of gene expression, especially for those proteins related to manganese oxidation such as MCO, which oxidizes Mn(II) into Mn oxides in *Pseudomonas* (Brouwers et al., 1999) and *Bacillus pumilus* (Su et al., 2013). However, the genes required to eliminate the toxicity of Mn(II) vary in the known MOBs. Moreover, for specific microorganisms that inhabit the soil in extreme environments, there is limited information available regarding their mechanisms of adaptation to high concentrations of Mn(II).

To investigate the adaptation mechanism of bacteria under Mn exposure, *B*. *safensis* strain ST7 was used for RNA-seq analysis of gene expression profiles in toxic Mn(II) media. A total of 3,755 genes from 12 cDNA libraries was detected, which included most genes in the reference transcriptome of *B. safensis*. There were 3,732 genes expressed in the exponential phase of growth, which decreased to 3,577 genes in the stationary phase. Mn(II) exposure specifically induced 64 genes and completely inhibited 53 genes in strain ST7. The genes induced by Mn(II) exposure in the exponential growth phase of strain ST7 were involved in the processes of transport and DNA repair, while the genes inhibited by Mn(II) exposure in this growth phase were involved in the processes of DNA replication and cell division. The patterns of specifically expressed genes might account for the slow growth of strain ST7 when encountering such a high concentration of Mn(II) (Supplementary Figure S7). Furthermore, in the stationary phase of growth, expression of genes related to sporulation was inhibited, while genes involved in membrane transport and secretion signaling were induced by Mn(II) exposure (Figure 3). This was similar to the profile of *Escherichia coli* MB266 obtained using iTRAQ-based proteomics analysis, whereby the proteins for ion and protein export and secretion systems were activated by Mn(II) (Wang et al., 2017).

It was recognized that 26 genes from the transcriptome of strain ST7were associated with lignocellulosic degradation (Supplementary Table S6). Lignocellulose in plant cell walls is composed of three components, namely cellulose, hemicellulose and lignin in the proportions of 35–50%, 25–35%, and 10–25%, respectively (Mazzoli, 2020). Cellulose is degraded into small molecules of chemicals mainly by cellobiohydrolases, endoglucanase, and β-glucosidases. Endoxylanase and β-xylosidase are two main enzymes involved in hemicellulose hydrolysis. The biodegradation of lignin requires oxidase and peroxidase, such as manganese peroxidase and laccase (Ma et al., 2020). Although no *Bacillus* members can ferment lignocellulosic material without prior pre-treatment of biomass saccharification, genes from ST7 transcriptome coding for cellulases, hemicellulases, and enzymes to decompose lignin were expressed at a high level according to the hierarchies of the KAAS enrichment analysis, and homology blasts with other *Bacillus* species (Kumar et al., 2016a; Ogonda et al., 2021). The other genes were related to the production of high-value chemicals such as ethanol, acetate, and lactate from cellulose. This indicated that strain ST7 has the potential to degrade lignocellulose while its capacity was inhibited by high concentrations of Mn(II).

### Differentially Expressed Genes Under Manganese Stress

Previous studies in yeast (Andreeva et al., 2017) and *Bacillus subtilis* (Mhatre et al., 2016) suggested that some DEGs based on RNA-Seq data may be associated with adaptation to excessive Mn(II). In the current study, 2,580 DEGs were identified in strain ST7. Many DEGs were highly expressed in ST7 upon Mn(II) exposure. These highly expressed genes might be pivotal to the basic processes for bacterial survival, such as carbohydrate and lipid metabolism, cellular community in quorum sensing, and transcription and translation, which would be necessary in the response to Mn stress. For instance, gene *RS09610* (encoding aconitate hydratase AcnA) was upregulated by Mn(II) with an increase greater than three-fold. AcnA modulates the efficiency of sporulation in *B. subtilis* (Craig et al., 1997), and a protein from marine spores can oxidize manganese (van Waasbergen et al., 1996). Thus, AcnA of ST7 might regulate the response to the toxic Mn(II) environment.

Gene *RS03010* of strain ST7 encoded an MCO-domain-containing protein belonging to the MCO family. The enzyme MCO catalyzes the transformation of Mn(II) into Mn(III/IV) (Bohu et al., 2015). Mn oxidation in bacteria is an important requirement for MOB to survive in extremely toxic environments. Mn oxidation in MOB indirectly expends ROS in the creation of Mn(II) oxide and relieves ROS stress on bacterial cells. Furthermore, biological Mn(III/IV) oxides can catalyze the degradation of refractory compounds into low-molecular-weight substrates as nutrients for bacteria and protect bacteria against toxic heavy metals such as arsenic (Sunda and Kieber, 1994; Tebo et al., 2005). The protein encoded by *RS03010* in strain ST7 showed 99% amino acid similarity (504/510) to the reference genome of *B. safensis* (Supplementary Figure S13).

Amino acid similarities of strain ST7 were much more diverse when compared with proteins from bacteria outside of the genus *Bacillus* that had been experimentally shown to harbor manganese oxidase (Corstjens et al., 1997; Brouwers et al., 1999; Hullo et al., 2001; Ridge et al., 2007; Dick et al., 2008; Geszvain et al., 2013; Su et al., 2013; Butterfield and Tebo, 2017). However, the four conserved domains for copper binding were identified in the MCO protein of strain ST7 (Supplementary Figures S13, S14). MCO, generally regarded as the indispensable enzyme in the process of Mn oxidation, resides on the outer surface of bacteria and accommodates four copper ions as cofactors (Su et al., 2013; Tang et al., 2021). The copper ions, which bind to conserved domains of MCO, are involved in electron transfer during the oxidation of Fe(II) or Mn(II) (van Waasbergen et al., 1996). Furthermore, in the experimental group with Mn(II) supplied at the exponential phase of growth, the expression level of the gene *RS03010* was 1.72-times higher than that in the absence of Mn(II) (Supplementary Table S4). When *RS03010* was knocked out, sporulation ceased, and the Mn oxidation capacity decreased to 26.22% in the mutant strain (Figure 7).

CotA, a spore-associated protein in the coat of prespores, is widely regarded as an MCO in *B. subtilis* (Hullo et al., 2001) and is stimulated under conditions of elevated temperature or pH (Baril et al., 2012). It was shown that proteins encoded by MCO genes from *Bacillus* sp. GZB, *B. pumilus* WH4, and *Brevibacillus panacihumi* MK-8 oxidize Mn(II) into Mn(IV) oxides using a heterologous expression system or site-directed mutagenesis method (Su et al., 2013; Das et al., 2020; Tang et al., 2021). These observations, together with the data from the current study, indicated that gene *RS03010* of strain ST7 is important for spore production and might encode the MCO protein needed to oxidize soluble manganese compounds. Bacterial MCOs are recognized as significant contributors to the Mn(II) oxidation process. Bacteria may have developed many strategies to adapt to environments containing extremely toxic quantities of manganese (Bai et al., 2021), and Mn(II) oxidation is a crucial mechanism used by MOB to facilitate survival in high Mn surroundings. The presence of Mn oxides outside of the bacteria provide protection against ultraviolet (UV) ionizing radiation, viral attack, and heavy metal toxicity (Daly et al., 2004; Ghosal et al., 2005).

Clusters of transporter genes with CPM > 1,000 were also upregulated by Mn(II) in the current study, based on KAAS enrichment analysis (Supplementary Table S5). Manganese transport systems encoded by *mntABCD* and *mntH* operons have been found from various bacteria such as *B. subtilis*, *Escherichia coli*, *Staphylococcus aureus*, *Salmonella enterica*, and *Corynebacterium diphtheria* (Kliegman et al., 2006). The *mntABCD* and *mntH* operons are controlled by manganese transport regulator MntR. However, except for *MntR*, these manganese transport genes were not annotated in the ST7 transcripts, and this may due to the imperfect annotation of the reference genome of *B. safensis* (assembly no. GCA_001895885.1). Clean data from the libraries were therefore realigned using *B. subtilis* strain 168 as a reference (GCA_000009045.1). Four genes, *mntB*, *mntD*, *mntH*, and *mntP*, were annotated in strain ST7, and all of them were markedly decreased under Mn(II) stress. Thus, the *mntABCD* and *mntH* systems might not function when *B. safensis* strain ST7 encounters high concentrations of toxic Mn(II). This suggests that different transporter systems in *B. safensis* might help extrude excessive Mn(II).

Nine genes in strain ST7 were annotated in transporter systems. Two of them were classified as ABC transporters in the cell membrane, including ABC transporter ATP-binding protein and iron ABC transporter permease (encoded by *RS16735*). Four of the nine genes were substrate-binding proteins (SBPs) that assist ABC transporters in the uptake of metal ions; these were ABC transporter substrate-binding protein, iron (3+)-hydroxamate-binding protein FhuD, iron-uptake system-binding protein, and heme ABC transporter substrate-binding protein IsdE. Amino acid permease (encoded by *RS17055*) predominantly uptakes amino acids into the bacteria cell, and some amino acid permeases are ABC-type transporters (Quintero et al., 2001).

ATP-binding cassette transporters comprise two domains—the intracellular nucleotide-binding domain (NBD) and the transmembrane domain (TMD) (Lorca et al., 2007). ATP binds with the NBD and provides energy, while the TMD provides a path for cargo to pass through the cell membrane. Additionally, ABC transporters require a SBP to carry the substrate outside the membrane and reach the TMD (Qasem-Abdullah et al., 2017). The mechanism of PTS transporters is markedly different from ABC-type and natural resistance-associated macrophage protein (NRAMP) transporters. The phosphoenolpyruvate-dependent carbohydrate transport system (a PTS encoded by gene *RS06145I* in strain ST7) performs the translocation together with concomitant phosphorylation of sugars, galactitol, and hexitols (Volpon et al., 2006).

The copper-binding protein encoded by gene *RS02135* in strain ST7 is the carrier that binds with Cu(I). Copper-binding protein, CopL, on the cell surface holds four Cu(I) ions on the outer surface of the cell and contributes to the Cu efflux effect of protein CopA or CopB (Rosario-Cruz et al., 2019). One copper-binding protein, prion protein (PrP), binds with both copper and manganese (Samorodnitsky and Nicholson, 2018). In the current study, the mutants with transporter genes *RS02135* and *RS17055* knocked out lost their Mn oxidation ability (Figure 7). Thus, these transporters might be manganese efflux pumps that remediate high concentrations of Mn(II), similar to the gene *mntE* in *Streptococcus mutans* (O’Brien et al., 2020) and *gsp* gene in *Pseudomonas putida* (de Vrind et al., 2003).

Several processes were significantly downregulated in strain ST7 under Mn exposure, including sporulation and flagellogenesis. In the sporulation pathway, the downregulated genes encoded enzymes and structural proteins required for spore assembly (Tables 2, 3). Species of the genus *Bacillus* produce dormant and metabolically inert spores to resist environmental stresses such as extreme pH and temperature conditions (Nicholson et al., 2000). The reported effects of Mn on bacterial sporulation are very diverse. Mn significantly enhanced sporulation in *B. cereus* (Ryu et al., 2005), but sporulation of *B. subtilis* strain B68 was completely suppressed under a Mn concentration of 7.2 mM/L (Song et al., 2007). This demonstrates that the regulation of sporulation is tightly controlled and diverse in bacteria.

Genes *RS08160* and *RS08165* in strain ST7 encode dipicolinic acid synthetase subunits A and B, which participate in the synthesis of dipicolinic acid (DPA) during the sporulation period in the mother cell, and DPA then accumulates in the dormant spores of *Bacillus* (Paidhungat et al., 2000). Genes *RS03530*, *RS10620*, and *RS10635* in strain ST7 encode glycosyltransferase, which catalyzes the transference and modification of monosaccharides in the crust during the late stage of sporulation (Shuster et al., 2019). Gene *RS15875* encodes KapD, which inhibits spore formation in *Bacillus thuringiensis* (Fan et al., 2017). The decrease in expression of these genes under Mn exposure could potentially block spore growth of strain ST7 in toxic Mn(II) media. Mutants Δ*RS10620* and Δ*RS10635* were completely defective in both sporulation and Mn(II) oxidation (Figure 7 and Supplementary Figure S11).

Although the relationship between glycosyltransferase and Mn has yet to be elucidated, it has been reported that spores from several species of the genus *Bacillus* contain proteins that oxidize Mn(II) into Mn(IV), including MnxG from the marine spores of *Bacillus* sp. SG-1 (Dick et al., 2008) and *Bacillus* sp. PL-12 (Butterfield and Tebo, 2017). In *B. subtilis*, a hallmark of the initiation of sporulation is the activation of Spo0A by phosphorylation through a phosphor-relay system (Rodriguez Ayala et al., 2020). When this system is activated by a starvation signal, a family of autophosphorylate kinases (KinA–KinE) initiates a cascade of phosphate transfers from sigma factor Spo0F to Spo0A. The production and accumulation of phosphorylated Spo0A (Spo0A-Pi) is the master regulator of sporulation. In addition, *AbrB* is a global gene regulator involved in the transition from exponential to stationary growth phase and antagonizes the sporulation process.

In the current study, *AbrB* was upregulated by Mn(II) in the stationary growth phase of strain ST7 (Supplementary Table S7), which inhibited the expression of *SigH* (Rodriguez Ayala et al., 2020). The SigH factor thus might not play a role in promoting the expression of both *Spo0F* and *Spo0A*. Furthermore, the high concentration of Mn(II) stimulated expression of *kipI* (logFC value 5.02), which then repressed the transcript of *kinA.* KinA is required for the phosphorylation and activation of Spo0F (Supplementary Table S7). In addition, the gene *rapA* was upregulated and is responsible for dephosphorylation of Spo0F-Pi (Rodriguez Ayala et al., 2020). These genes ultimately act to decrease the accumulation of Spo0A-Pi so that fewer spores are produced and sporulation is much slower in the harsh Mn(II) environment compared with the control environment lacking Mn(II).

Another stress-related pathway, the SigB pathway, was upregulated at the start of the stationary growth phase in strain ST7. It was reported that spore formation is the final survival strategy because it is a process that consumes large quantities of energy, and spore germination and outgrowth need to be tightly regulated to recover the planktonic growth in *B. subtilis* (Rodriguez Ayala et al., 2020). Furthermore, the SigB pathway is a link that interconnects the two dominant and mutually exclusive adaptive responses—sporulation and general stress response (GSR)—in the surviving regulatory network of *B. subtilis* and is needed for the bacteria to cope with very harsh conditions (Rodriguez Ayala et al., 2020).

Similar to *B. subtilis*, the *SigB* gene was upregulated 2.85-times in strain ST7 cultured in Mn(II)-supplemented media (Supplementary Table S7). The SigB pathway participates in regulation of the stress response under extreme conditions such as heat, salt, pH, and manganese in a *B. subtilis* model (Rodriguez Ayala et al., 2020). Congruent with this, a high concentration of Mn(II) in the culture media induced expression of the kinase *RsbT* in strain ST7, which may phosphorylate RsbR analogs and RsbS (Supplementary Table S7). The released RsbT protein can bind another phosphatase, RsbU, to dephosphorylate RsbV-Pi (Rodriguez Ayala et al., 2020). Dephosphorylated RsbV then binds to RsbW, releasing SigB, which in turn combines with RNA polymerase and activates its target genes. For example, the mRNA level of *ctc* (the general stress protein, similar to ribosomal protein L25) was upregulated approximately 7.39-fold in strain ST7 exposed to Mn(II) (Supplementary Table S7).

In the flagellum pathway, 32 genes were upregulated in strain ST7 at the exponential phase of growth following Mn exposure, but only 10 flagellum genes were expressed at the stationary phase of growth, and nine of these genes were downregulated (Table 5). The flagellar structure of *Bacillus* is complex and contains three basic structures—the hook, the basal body, and the long filament (Terahara et al., 2020). Of the 32 genes upregulated by Mn exposure in strain ST7 at the exponential phase of growth, genes encoding structural proteins were expressed at a high level. The proteins encoded by these genes were necessary for the assembly of flagella, including FlaD and flagellar capping protein located in the hook; MotA and MotB in the stator; FliD, FlgK, and FlgL in the filament; FlgB, FlgC, FliE, and FliF in the basal body; FliG in the rotor; and FliM and FliY, which are used to control the movement direction of flagellum.

**TABLE 5:** Flagellar genes expressed in exponential and stationary growth phases of strain ST7 exposed to manganese.

No.	Gene_ID	logFC	Up/down	Product	Path/hierarchy	Mean (Mn (II))	Mean (Control)
**Exponential phase**							
1	RS07920	3.95	up	flagellar motor switch protein FliM	Flagellar assembly path [ko02040]; Chemotaxis path [ko02030]	8425.00	544.64
2	RS07940	4.04	up	flagellar type III secretion system pore protein FliP	Flagellar assembly path [ko02040]	3484.05	211.39
3	RS07950	3.94	up	flagellar type III secretion system protein FliR	Flagellar assembly path [ko02040]	3361.18	218.42
4	RS07935	3.94	up	flagella biosynthesis regulatory protein FliZ	Flagellar assembly path [ko02040]	4412.17	287.39
5	RS07905	3.56	up	flagellar basal body rod protein FlgG	Flagellar assembly path [ko02040]	5783.42	491.55
6	RS07900	3.86	up	flagellar hook assembly protein FlgD	Flagellar assembly path [ko02040]	2812.57	193.89
7	RS07915	3.74	up	flagellar basal body-associated protein FliL	Motility hierarchy [BR:ko02035]	2926.04	219.84
8	RS07925	4.05	up	flagellar motor switch phosphatase FliY	Flagellar assembly path [ko02040]; Chemotaxis path [ko02030]	7159.87	433.39
9	RS07880	3.39	up	flagellar protein export ATPase FliI	Flagellar assembly path [ko02040]	7395.22	707.71
10	RS07965	3.51	up	flagellar biosynthesis protein FlhF	Motility hierarchy [BR:ko02035]	2818.12	246.68
11	RS07885	3.30	up	flagellar biosynthesis chaperone FliJ	Flagellar assembly path [ko02040]	1791.89	181.42
12	RS07850	2.90	up	flagellar basal body rod protein FlgB	Flagellar assembly path [ko02040]	2442.03	326.97
13	RS07895	3.54	up	flagellar hook-length control protein	Flagellar assembly path [ko02040]	7398.99	637.55
14	RS07860	3.09	up	flagellar hook-basal body complex protein FliE	Flagellar assembly path [ko02040]	1836.34	216.19
15	RS07865	3.06	up	flagellar basal body M-ring protein FliF	Flagellar assembly path [ko02040]	12472.03	1493.58
16	RS07875	3.28	up	flagellar assembly protein FliH	Flagellar assembly path [ko02040]	4168.67	428.55
17	RS07960	2.62	up	flagellar biosynthesis protein FlhA	Flagellar assembly path [ko02040]	4093.97	667.79
18	RS07855	2.93	up	flagellar basal body rod protein FlgC	Flagellar assembly path [ko02040]	2093.31	275.11
19	RS07870	2.87	up	flagellar motor switch protein FliG	Flagellar assembly path [ko02040]; Chemotaxis path [ko02030]	6746.57	923.08
20	RS17660	2.22	up	flagellar hook-associated protein FlgL	Flagellar assembly path [ko02040]	1314.91	283.02
21	RS07945	3.01	up	flagellar biosynthesis protein FliQ	Flagellar assembly path [ko02040	549.35	67.78
22	RS17635	2.04	up	flagellar hook-associated protein 2	Flagellar assembly path [ko02040]	569.16	138.34
23	RS17630	1.96	up	flagellar export chaperone FliS	Flagellar assembly path [ko02040]	463.71	119.23
24	RS17625	1.67	up	flagellar protein FliT	Flagellar assembly path [ko02040]	588.10	184.86
25	RS06050	2.45	up	flagellin Hag	Flagellar assembly path [ko02040]	1500.93	273.93
26	RS17665	1.74	up	flagellar hook-associated protein FlgK	Flagellar assembly path [ko02040]	2232.71	670.15
27	RS17670	1.42	up	flagellar protein FlgN		549.07	204.85
28	RS06550	1.26	up	flagellar motor protein MotB	Flagellar assembly path [ko02040]; Chemotaxis path [ko02030	742.19	309.18
29	RS06555	1.64	up	flagellar motor stator protein MotA	Flagellar assembly path [ko02040]; Chemotaxis path [ko02030]	668.15	214.81
30	RS18115	1.04	up	flagellar hook-basal body protein	Flagellar assembly path [ko02040]	2967.46	1442.44
31	RS06055	1.00	up	flagellin Hag	Flagellar assembly path [ko02040]	227.78	114.28
32	RS17675	1.15	up	flagellar biosynthesis anti-sigma factor FlgM	Flagellar assembly path [ko02040]	347.85	156.92
33	RS17640	0.97	no-diff	flagellar protein FlaG	Motility hierarchy [BR:ko02035]	206.93	105.22
Stationary growth phase							
1	RS06555	−3.74	down	flagellar motor stator protein MotA	Flagellar assembly path [ko02040]; Chemotaxis path [ko02030]	180.24	214.81
2	RS17635	−2.23	down	flagellar hook-associated protein 2	Flagellar assembly path [ko02040]	79.06	138.34
3	RS17630	−2.09	down	flagellar export chaperone FliS	Flagellar assembly path [ko02040]	84.17	119.23
4	RS06550	−2.04	down	flagellar motor protein MotB	Flagellar assembly path [ko02040]; Chemotaxis path [ko02030]	277.78	309.18
5	RS17625	−1.76	down	flagellar protein FliT	Flagellar assembly path [ko02040]	65.14	184.86
6	RS07935	−1.74	down	flagella biosynthesis regulatory protein FliZ	Flagellar assembly path [ko02040]	1111.23	287.39
7	RS07945	−1.71	down	flagellar biosynthesis protein FliQ	Flagellar assembly path [ko02040]	83.72	67.78
8	RS07925	−1.57	down	flagellar motor switch phosphatase FliY	Flagellar assembly path [ko02040]; Chemotaxis path [ko02030]	1490.87	433.39
9	RS07880	−1.35	down	flagellar protein export ATPase FliI	Flagellar assembly path [ko02040]	1841.29	707.71
10	RS05910	2.00	up	flagellin Hag	Flagellar assembly path [ko02040]	1290.59	277.32

The functions of flagella change with the growth states of bacteria. *B. subtilis* exists in two states, including cell chains joined end-to-end and a single bacterium moving alone during the exponential growth phase (Chai et al., 2010). Flagellar genes of *B*. *subtilis* are expressed in the exponential growth phase, while the sessile biofilm-forming-related genes are induced in the stationary phase, during which the transition from motile cells to sessile growth is controlled by phosphorylation of modulator *degU* (Kobayashi, 2007). In strain ST7, swimming motility was stimulated but swarming motility was not affected by Mn(II) (Supplementary Figure S6). The gene *RS17705* in strain ST7, corresponding to *degU*, was upregulated in the exponential phase of growth but was not detected in the stationary phase. Additionally, the biofilm formation of ST7 was promoted by Mn(II). This suggested that motility might be controlled by the *degU* gene of strain ST7. Furthermore, it has been reported that Mn(II) exposure increases the expression of flagellar genes (e.g., *fliI*, encoding the sigma factor for the flagellar operon) and the motility of gut bacteria in mice (Chi et al., 2017). Interestingly, disruption of Mn oxidation and a decrease in motility was observed in mutants Δ*RS07865* (encoding FliF), Δ*RS07895* (encoding flagellar hook-length control protein), Δ*RS07920* (encoding FliM), and Δ*RS07980* (encoding chemotaxis protein CheA) in the current study (Figure 7). This confirmed that expression of flagellar genes directly affected the capacity for Mn oxidation in strain ST7.

## Conclusion

The capacities for Mn removal by the bacterial strains isolated from soil originating from a Mn mine area were very diverse. *B. safensis* strain ST7 was the most effective MOB among the isolated strains. The adaptation mechanism in *B*. *safensis* strain ST7 when confronted by an environment with a high concentration of Mn(II) is multifaceted, involving extracellular precipitation, enzymatic oxidation, and active efflux pumps. Increased expression of an MCO gene to oxidize excessive Mn(II), stimulation of transporter genes to pump excess Mn outside of the cell, and inhibition of expression of sporogenesis genes and flagellar genes to save energy and direct flux were detected. Many metabolic processes were also significantly affected by exposure to Mn. It was determined that an alternative stress-related pathway, the SigB pathway, was upregulated by Mn(II) at the stationary growth phase of strain ST7. The multi-metal tolerance traits of the isolated strain coupled with the high removal rate of Mn suggest that strain ST7 could be an effective bioremediation candidate for the restoration of ecological environments damaged by Mn and other heavy metal pollutants. The use of such a bacterial strain for bioremediation minimizes the use of chemical oxidants and is a potentially cost-effective technology. Additionally, the strain ST7 might degrade lignocellulosic biomass with other microbial collaborators to produce high-value chemicals with high efficiency (such as ethanol, acetate, and lactate) through the microbial fermentation pathway as an alternative to petroleum-based routes.

## Data Availability Statement

The sequencing data for this study can be found in the SRA database of National Center for Biotechnology Information (NCBI) (Bioproject number PRJNA734365). The nucleotide sequence of 16S rDNA and gyrase subunit A (*gryA*) gene of strain ST7 are available in the NCBI under accession number of MT378374 and MT449449.

## Author Contributions

XR, SL, and JW designed the work and the experiments. HL, QT, ZZ, LY, XW, QL, SH, and XN performed the experiments and data analyses. XR analyzed the data and wrote the manuscript. JW revised the manuscript. All authors contributed to the article and approved the submitted version.

## Conflict of Interest

The authors declare that the research was conducted in the absence of any commercial or financial relationships that could be construed as a potential conflict of interest.

## Publisher’s Note

All claims expressed in this article are solely those of the authors and do not necessarily represent those of their affiliated organizations, or those of the publisher, the editors and the reviewers. Any product that may be evaluated in this article, or claim that may be made by its manufacturer, is not guaranteed or endorsed by the publisher.

## Footnotes

1
https://github.com/OpenGene/fastp

2
https://github.com/alexdobin/STAR

3
http://www.r-project.org/

4
http://sangerbox.com/Tool

5
https://www.genome.jp/tools/kaas/
