# Supplementary material for: Manganese Stress Adaptation Mechanisms of Bacillus safensis Strain ST7 From Mine Soil
Source: Front Microbiol. 2021 Nov 25;12:758889. doi: 10.3389/fmicb.2021.758889 (PMC8656422; doi:10.3389/fmicb.2021.758889)
Supplement: Supplementary file 1 [file Image_1.pdf]

Supplementary Figures

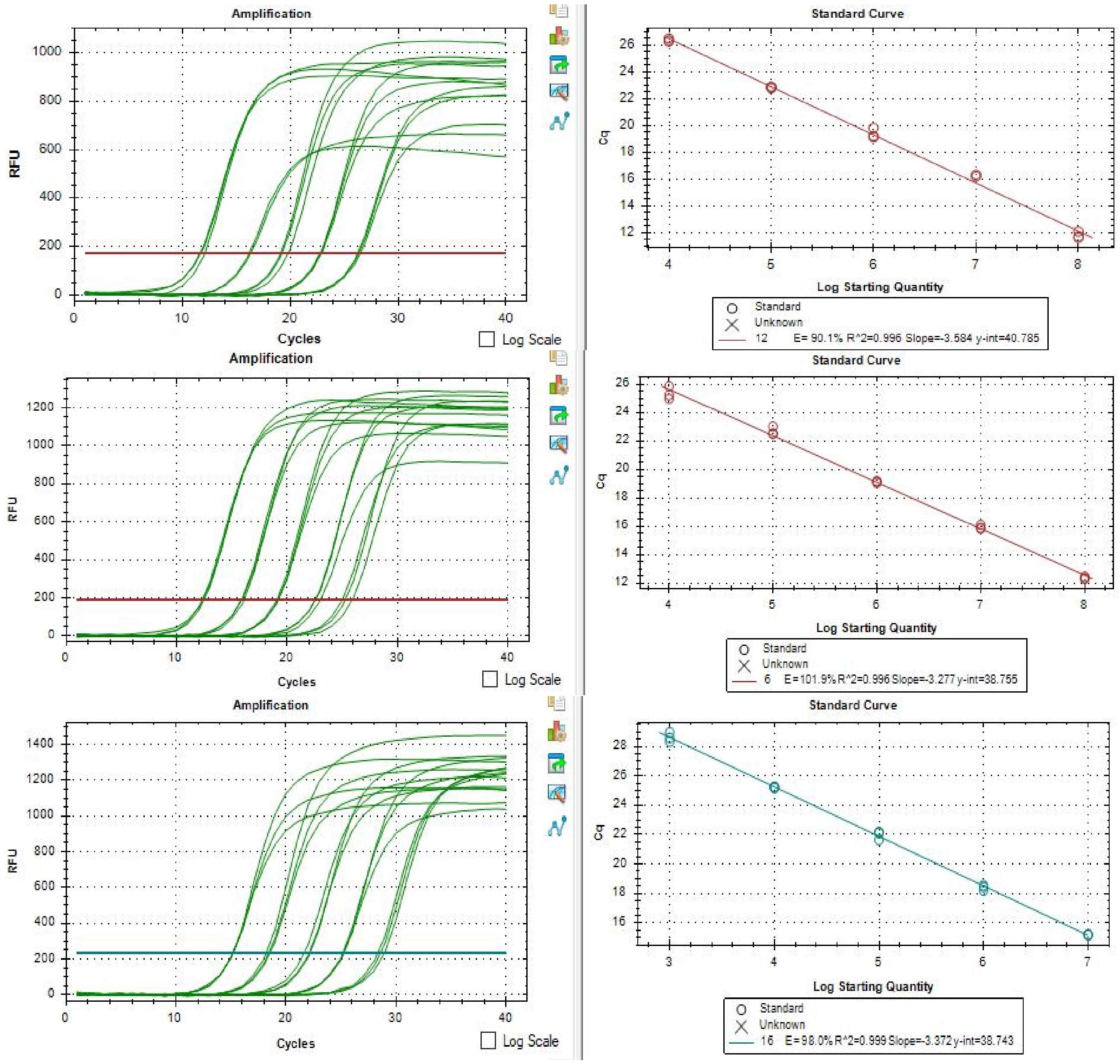

Supplementary Figure S1. The amplification and standard curves for representative transcripts taking the positive recombined plasmids as templates by RT-qPCR. The efficiency was denoted at the bottom of standard curve diagram with templates to be serially diluted by ten times of gradients.

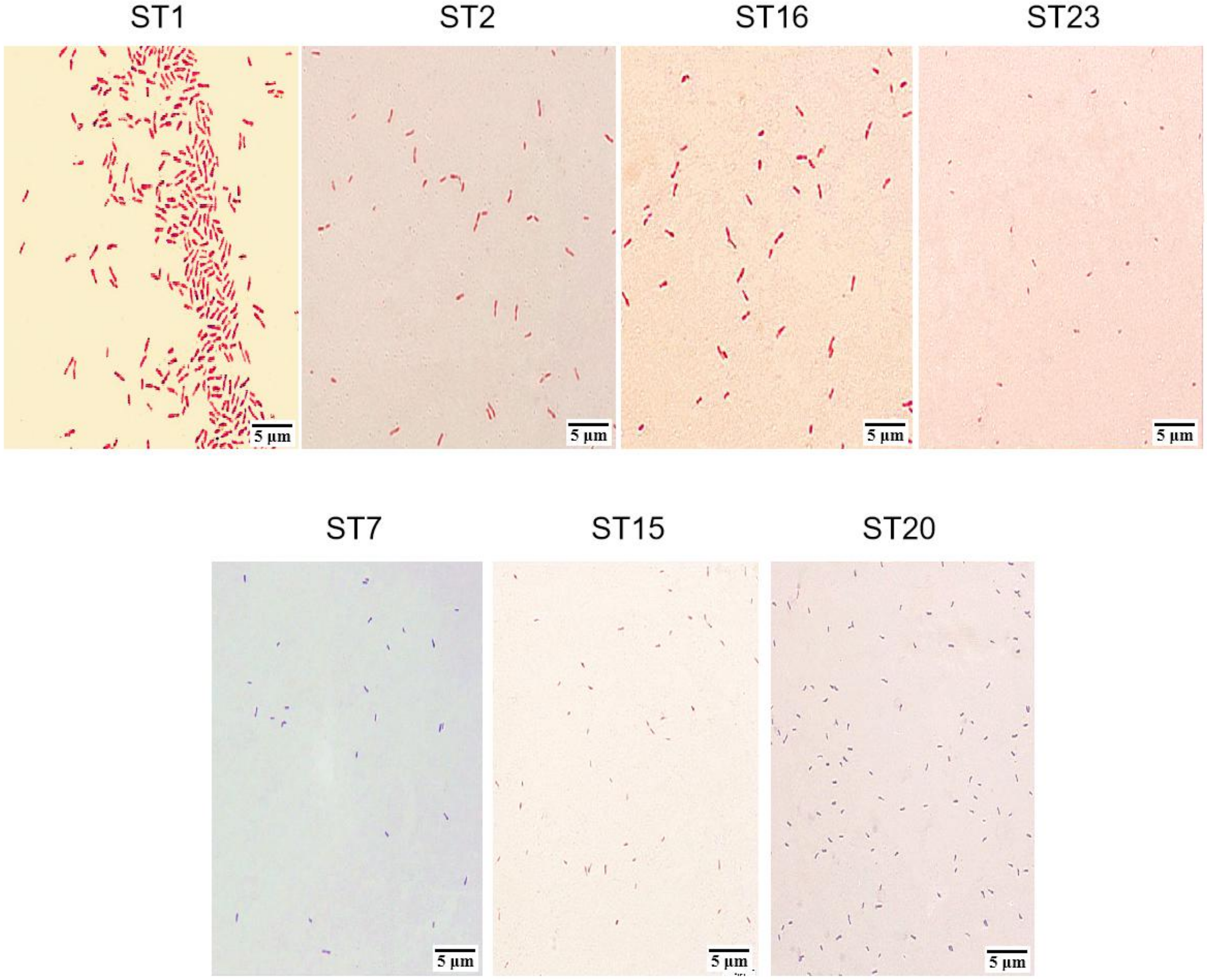

Supplementary Figure S2. The morphology and Gram staining of partial isolated strains from soil.

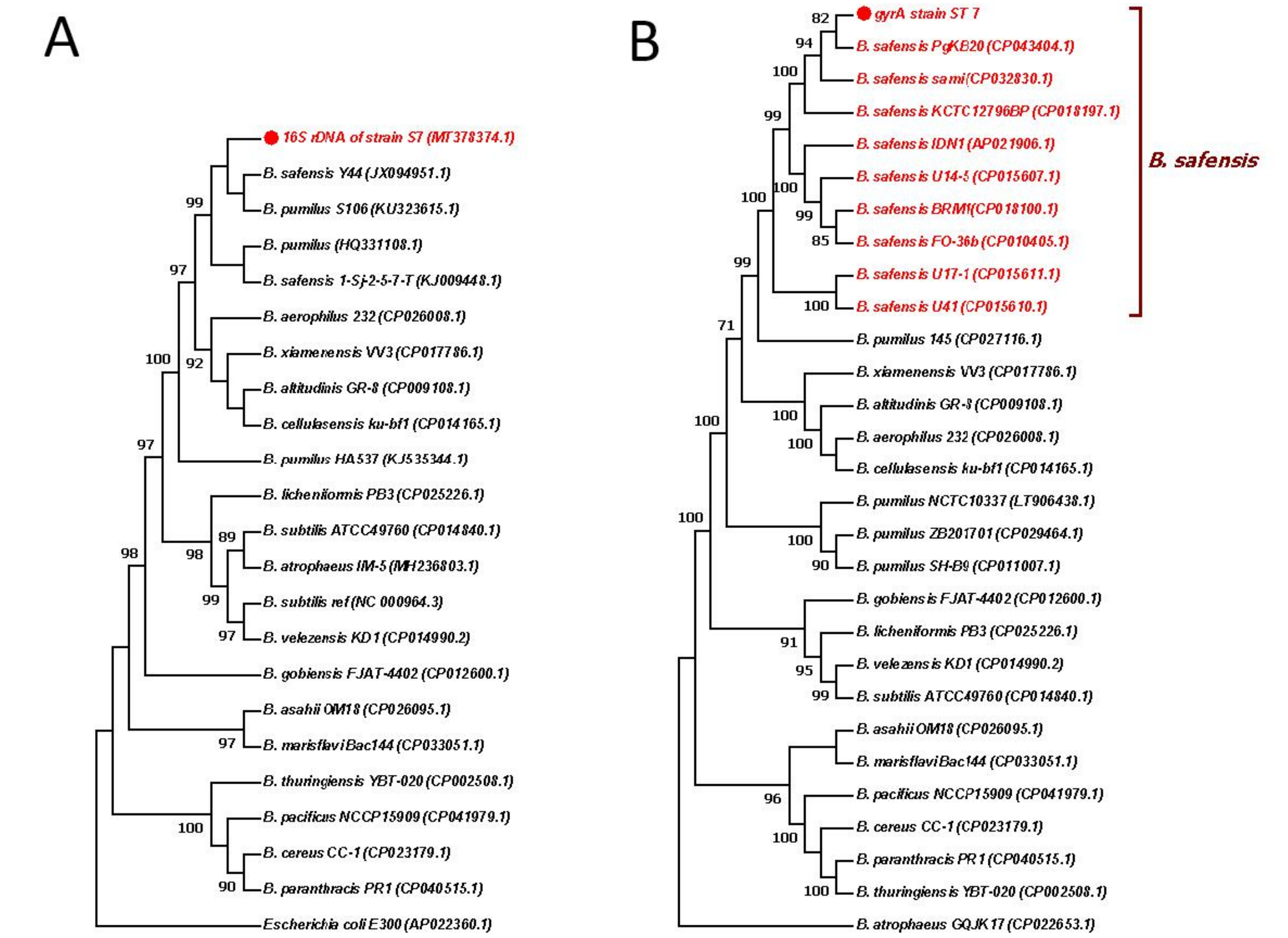

Supplementary Figure S3. The phylogenetic trees of 16S rRNA and *gryA* genes of *B. safensis* strain ST7 constructed by MEGA7.

The molecular phylogenetic analysis of genes were inferred by using the UPGMA method based on the Tamura 3-parameter model corrected by gamma distribution. **A**: the phylogenetic tree based on the nucleotide sequence of 16S rRNA gene. **B**: the phylogenetic tree of DNA gyrase subunit A (*gryA*) gene.

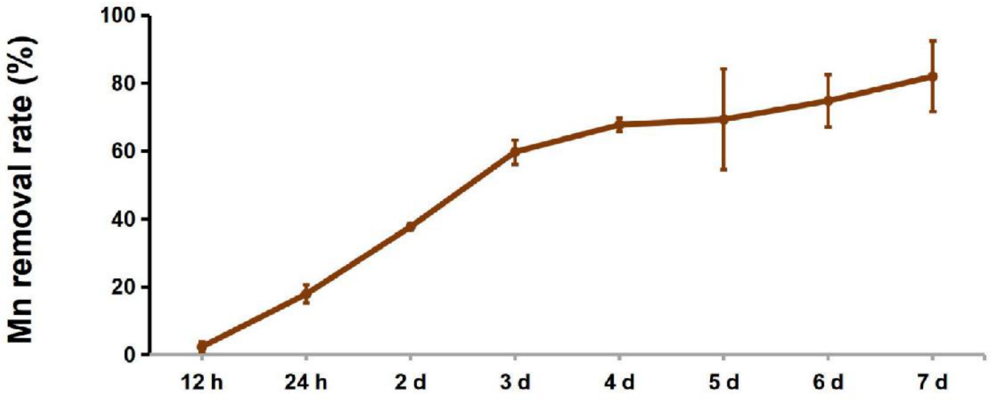

Supplementary Figure S4. The Mn (II) absorption efficiencies of strain ST7 from day 1 to day 7.

The bacteria were cultivated from 1 day to 7 days in PYCM media with 250 mg/L $MnCl_2$. The Mn(II) absorption efficiency was tested by ICP-OES. The Mn absorption efficiencies were increased in a time-dependent manner with the higher efficiencies started from day 3 and hold up to day 7.

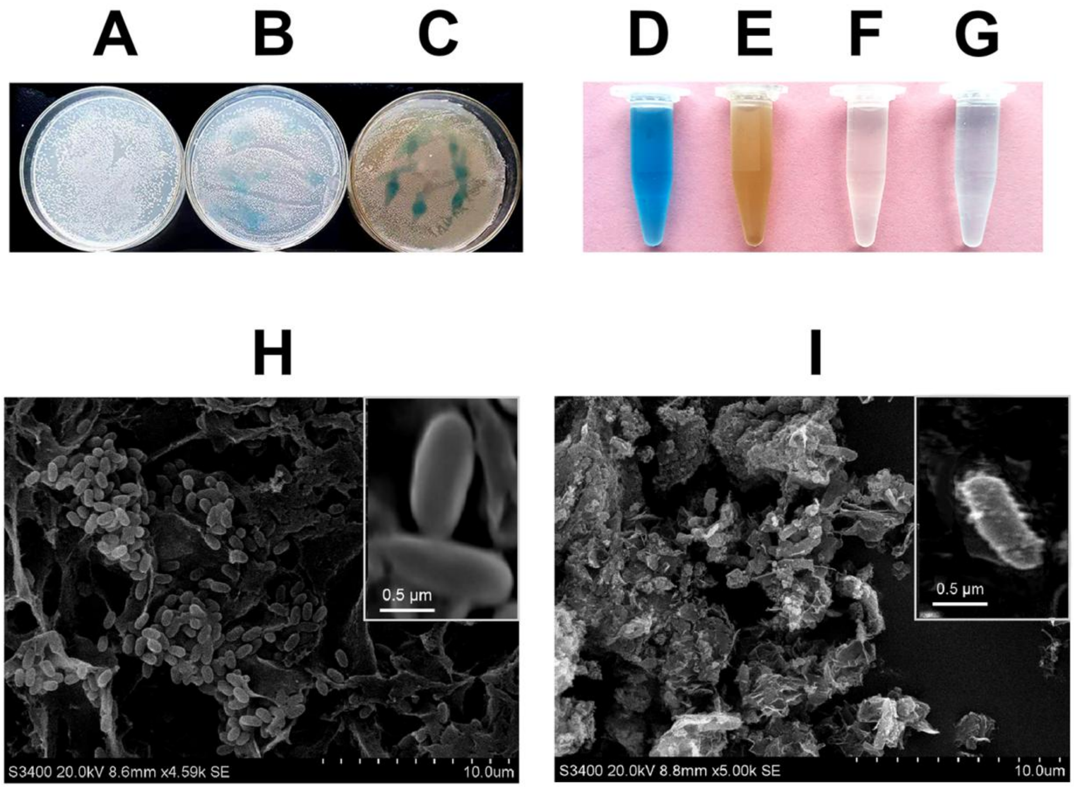

Supplementary Figure S5. Observation of the Mn (II) oxidation in strain ST7 by LBB solution and SEM.

The colonies of strain ST7 were incubated for seven days at 28˚C. About 150 μL 0.04% LBB were spread onto each plate. **A**: Strain ST7 cultured on PYCM solid plate. **B**: Strain ST7 cultured on PYCM plate with 250 mg/L $MnCl_2$. It produced Mn oxides in faint blue colors dyed by LBB. **C**: Strain ST7 cultured on PYCM plate with 2200 mg/L $MnCl_2$. Several stripes in deep blue colour could be observed on the plate. **D**: The supernatants of ST7 cultures supplied with 250 mg/L $MnCl_2$ was stained by LBB solution into deep blue color. **E:** The cultures of strain ST7 with 250 mg/L $MnCl_2$ presented in brown color. Both of ST7 cultures (**F**) and the LBB solution (**G**) were nearly colorless. The precipitates of manganese oxides outside of bacterium cultivated for seven days in PYCM liquid media without Mn(II) (**H**) or supplied with 2200 mg/L $MnCl_2$ (**I**) were observed by SEM.

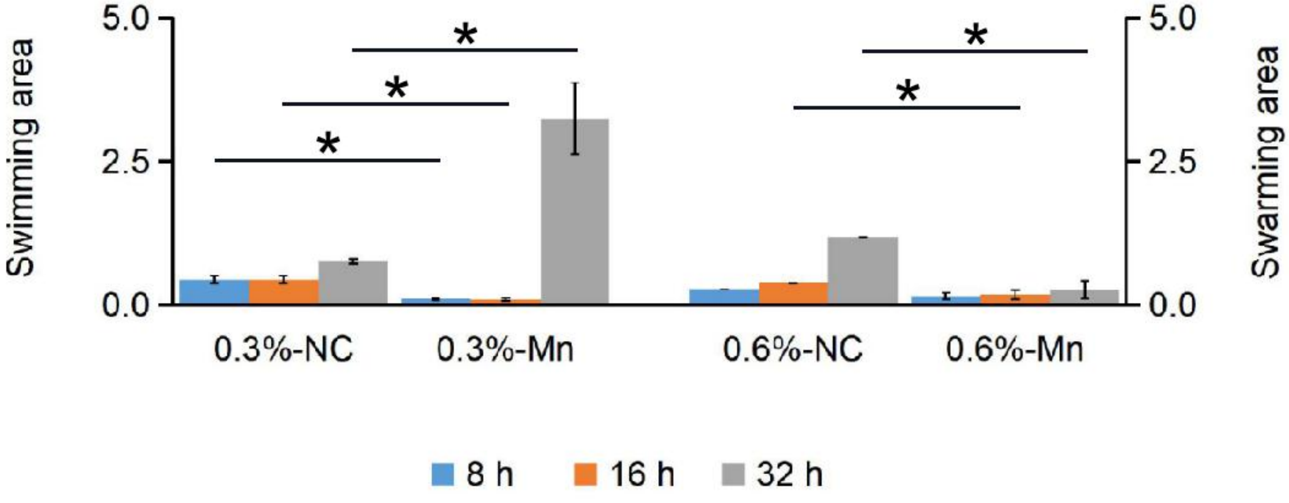

Supplementary Figure S6. The motility of strain ST7 under Mn stress.

The swimming and swarming motility were detected in PYCM plates with 0.3 % and 0.6 % soft-agar. The plates were punctured with 2 μL of strain ST7 cultured in LB media at 28 °C for 8 hours. Then, the plates were incubated at 28 °C for 8-32 hours, which were repeated in four times. Compared with control plates (NC) without Mn(II) supply, the area of bacterial colony in 250 mg/L Mn(II) was smaller in 0.6% agar plates cultured for 8-32 h and 0.3% agar plates for 8-16 h. But it was obviously stimulated by Mn(II) in 0.3% agar plates cultured for 32 h (grey column).

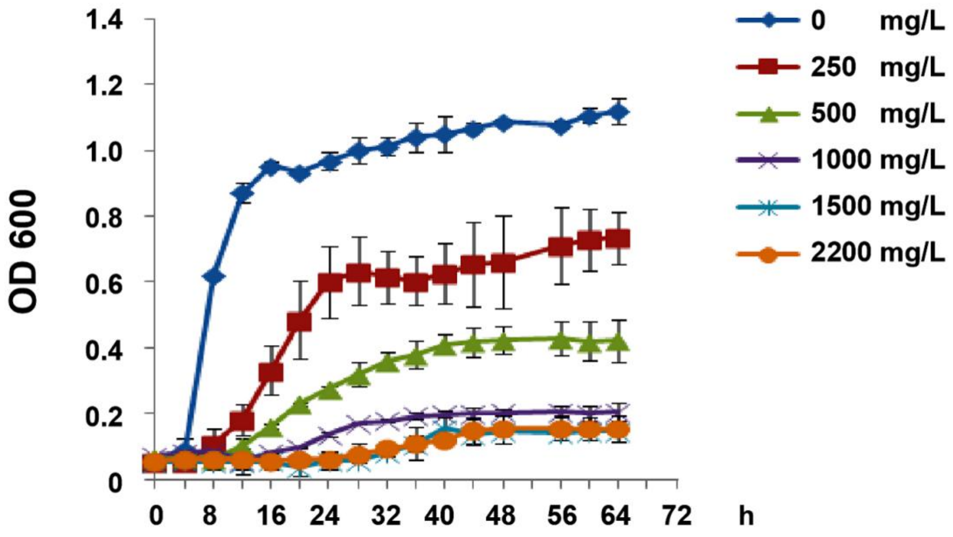

Supplementary Figure S7. The growth curves of strain ST7 at different concentrations of manganese stress.

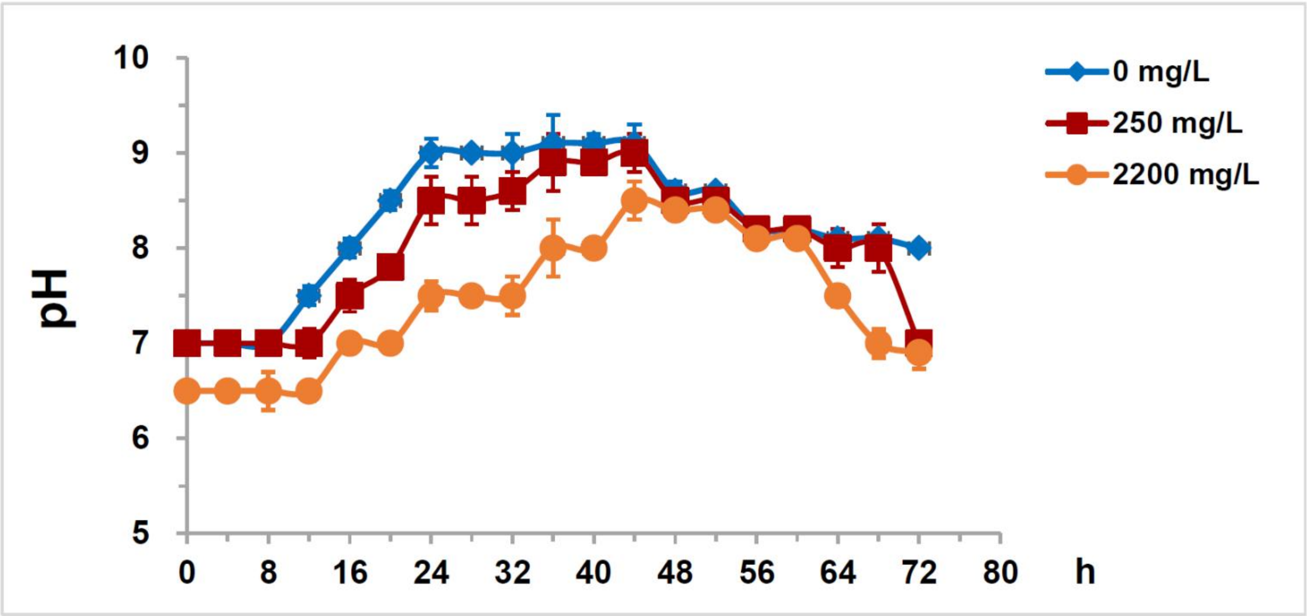

Supplementary Figure S8. The pH changes in the cultures of strain ST7 at different concentrations of manganese stress.

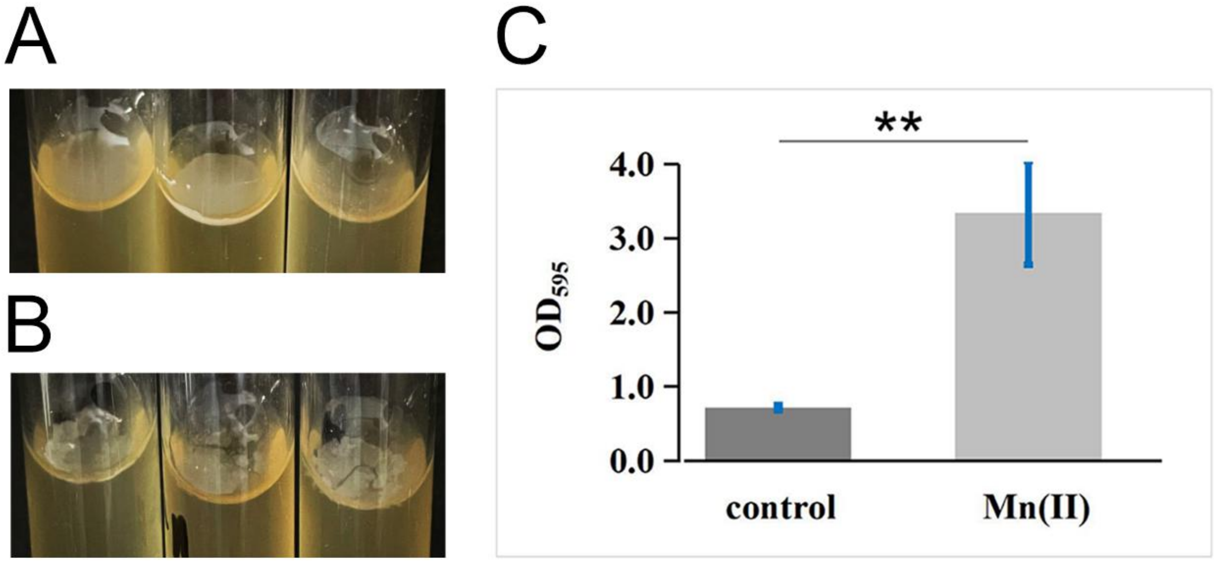

Supplementary Figure S9. Effects of manganese on biofilm formation of *B. safensis* strain ST7

A: The biofilm of *B. safensis* strain ST7 in LB media cultured for 72 h. **B**: The biofilm of strain ST7 in LB media supplemented with 250 mg/L Mn(II) for 72 h. **C**: The absorbance at 595 nm (OD_{595}) of solubilized crystal violet from microtiter plate assay detected as previously described (Auger et al, 2006).

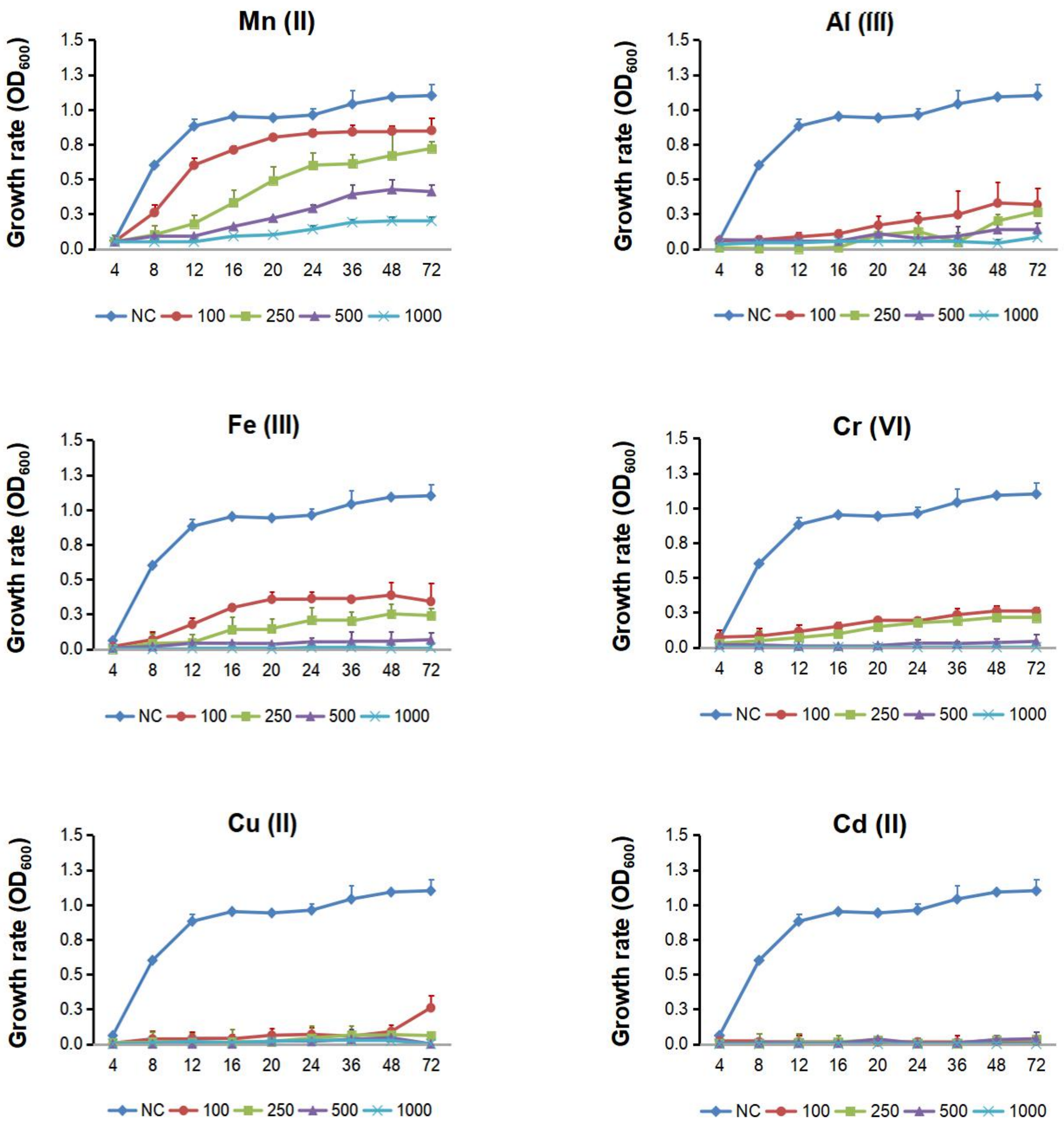

Supplementary Figure S10. The growth of *B. safensis* strain ST7 in media supplied with heavy metals.

The isolated strain ST7 were inoculated in PYCM media supplied with heavy metals at varied concentrations. The bacteria could grow and tolerate Al(III) in 500 mg/L, Cr(VI) and Fe(III) in 250 mg/L, but it could not grow in media contained Cd(II) and Cu(II).

A B C D E F

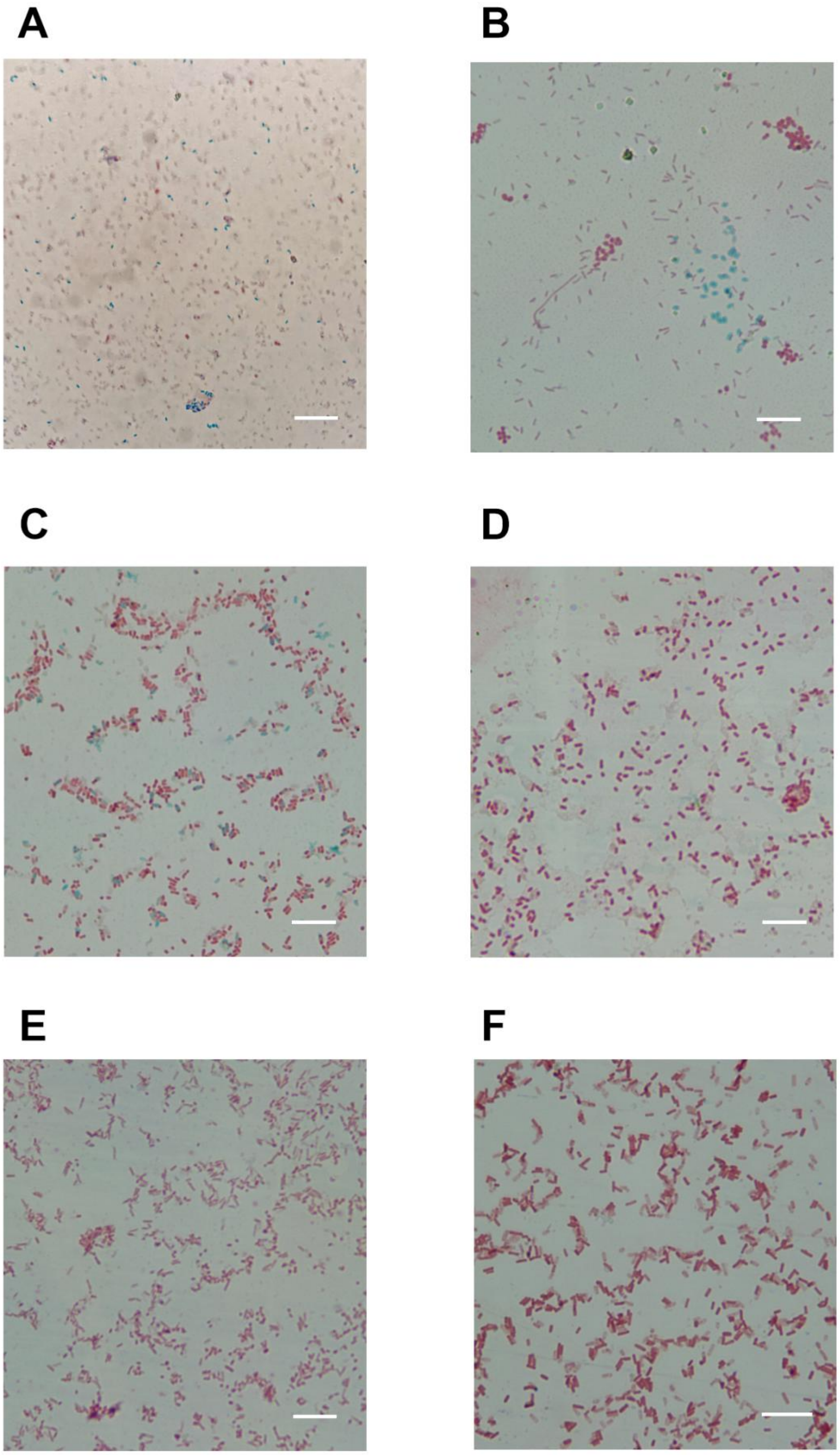

Supplementary Figure S11. The spores of mutants with sporulation genes knocked out stained by malachite green.

Strain ST7 and the mutants were cultured in PYCM broth for 72 h and stained by malachite green and safranin solution (Spore staining kit, Solarbio, Shanghai). Endospore in bacteria was stained in blue color. **A**: wild strain ST7 with spore percentage of 51%. **B**: mutant ΔRS11215 with spore of 37%. **C**: mutant Δ*RS05775* with spore of 31%. The spore could not be observed in three mutants of **D**: Δ*RS10635*. **E**: Δ*RS10620*. **F**: Δ*RS03010*. Bar=5 μm.

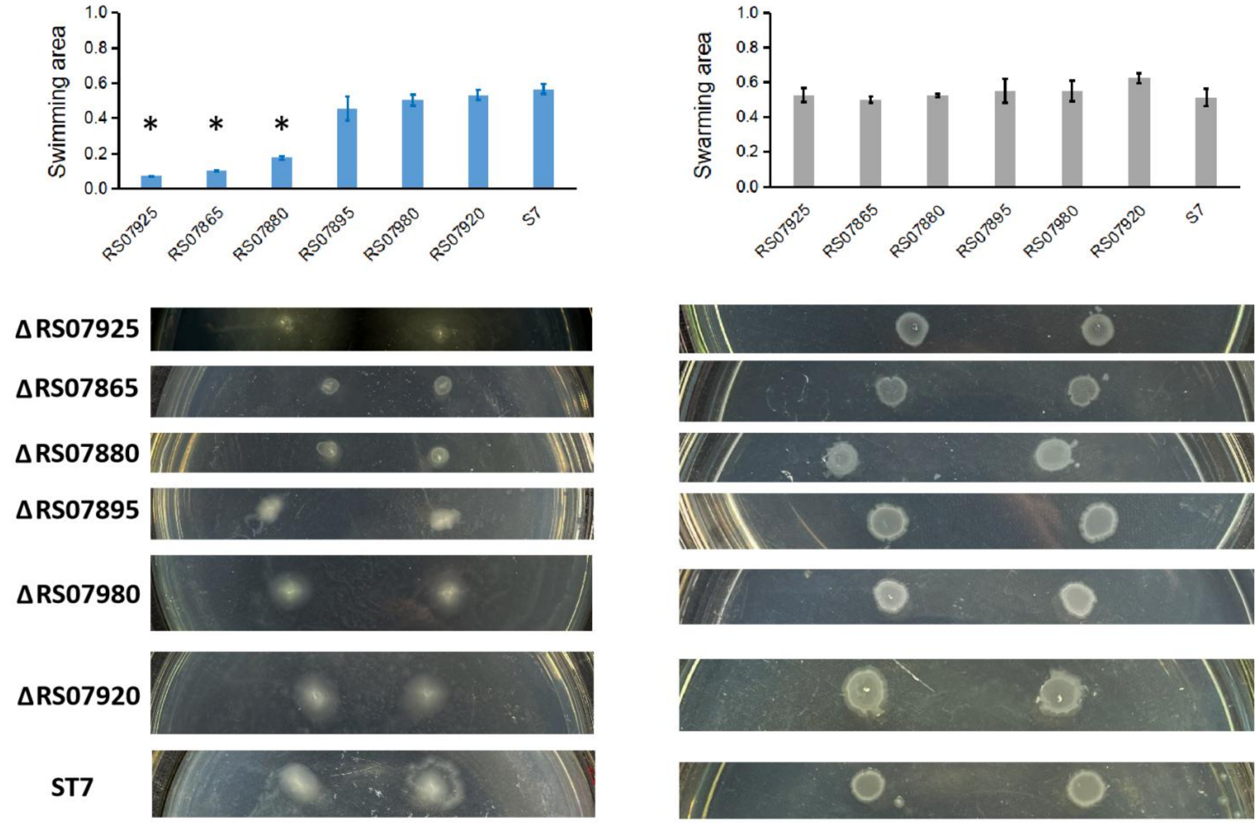

Supplementary Figure S12. The motility of mutants with flagellar genes knocked out.

The swimming and swarming motility of mutants were detected by soft-agar plates of PYCM media cultured for 24 hours. The swimming capacities of three mutants (Δ*RS07925*, Δ*RS07865* and Δ*RS07880*) were decreased, the swarming abilities were similar with the wild strain ST7.

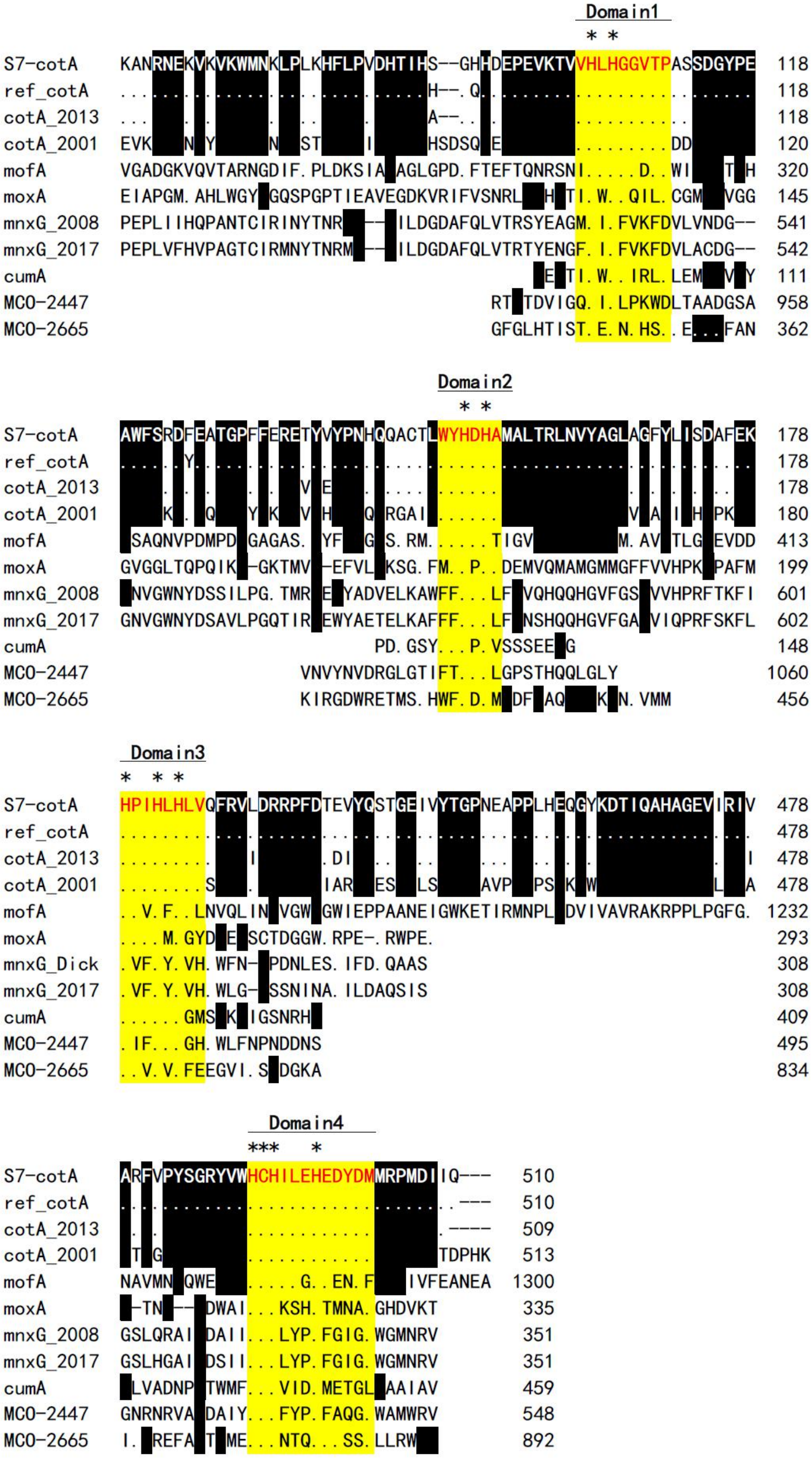

Supplementary Figure S13. Compare of amino acids sequences in the domain regions of MCOs coded by gene RS03010 in strain ST7 with those of other known MOBs.

The possible copper-binding domains were depicted in yellow blocks (domain 1 to 4) and the conserved amino acids surrounding the domains are shaded in black color. The conserved residues were indicated by asterisks. The putative copper-binding motifs in the MCOs of strain ST7 were assigned to range from residues 102 to 110, 149 to 154, 419 to 426, and 491 to 502. **ST7-MCO:** coded by RS03010 of strain ST7 in the present paper. The "**ref-MCO**" is the multicopper oxidase domain-containing protein (WP_073204070.1) coded by gene RS03010 in the reference genome of *Bacillus safensis* deposited in NCBI with assembly number GCF_001895885.1. The "**cotA_2013**" (AFL56752.1) is the cotA of *Bacillus pumilus* strain WH4 (Su et al., 2013). "**cotA_2001**" is the cotA (NP_388511.1) of *Bacillus subtilis*_strain 168 reported by Hullo (2001). "**mofA**" (CAA81037.2) is from *Leptothrix discophora* strain SS-1 (Corstjens et al., 1997). "**moxA**" (CAJ19378.1) is reported from *Pedomicrobium* sp. strain ACM3067 (Ridge et al., 2007). "**mnxG_2008**" (AAB06489.1) is identified from *Bacillus* sp. strain SG-1 isolated from marine sediments (Dick et al., 2008). "**mnxG_2017**" (EF158106) is identified from *Bacillus* sp. PL-12 isolated from marine (Butterfield and Tebo, 2017). "**cumA**" (AAD24211.1) is from *Pseudomonas putida* GB-1 (Brouwers et al., 1999). "**MCO-2447**" and "**MCO-2556**" are recognized to be the multicopper oxidase coded by gene2447 (ABY98346.1) and gene2665 (ABY98562.1) of *Pseudomonas putida* GB-1, respectively (Geszvain et al., 2013). The dashes represent gaps in the alignment and dots indicated the same residues with the sequences at first line.

The coded MCO protein of strain ST7 present 98% of similarity (1497/1533) in nucleotides, and 99% similarity (504/510) in amino acids with the record of the reference gene deposited in NCBI. The similarities of cotA between strain ST7 and the other *Bacillus* cotA enzymes were much higher while it fall down rapidly if compared with other proteins outside *Bacillus* with manganese oxidase capacity confirmed by experiments. It is 95.3% (486/510) identity to the cotA of *Bacillus pumilus* strain WH4 reported by Su et al (2013), 69% (353/514) identity to the cotA from *Bacillus subtilis* strain 168 (NP_388511.1) (Hullo et al., 2001). The length of proteins with Mn(II) oxidation activity in other bacteria are quite diverse from that of *Bacillus* species (Corstjens et al., 1997; Brouwers et al., 1999; Ridge et al., 2007; Dick et al., 2008; Geszvain et al., 2013; Butterfield and Tebo, 2017).

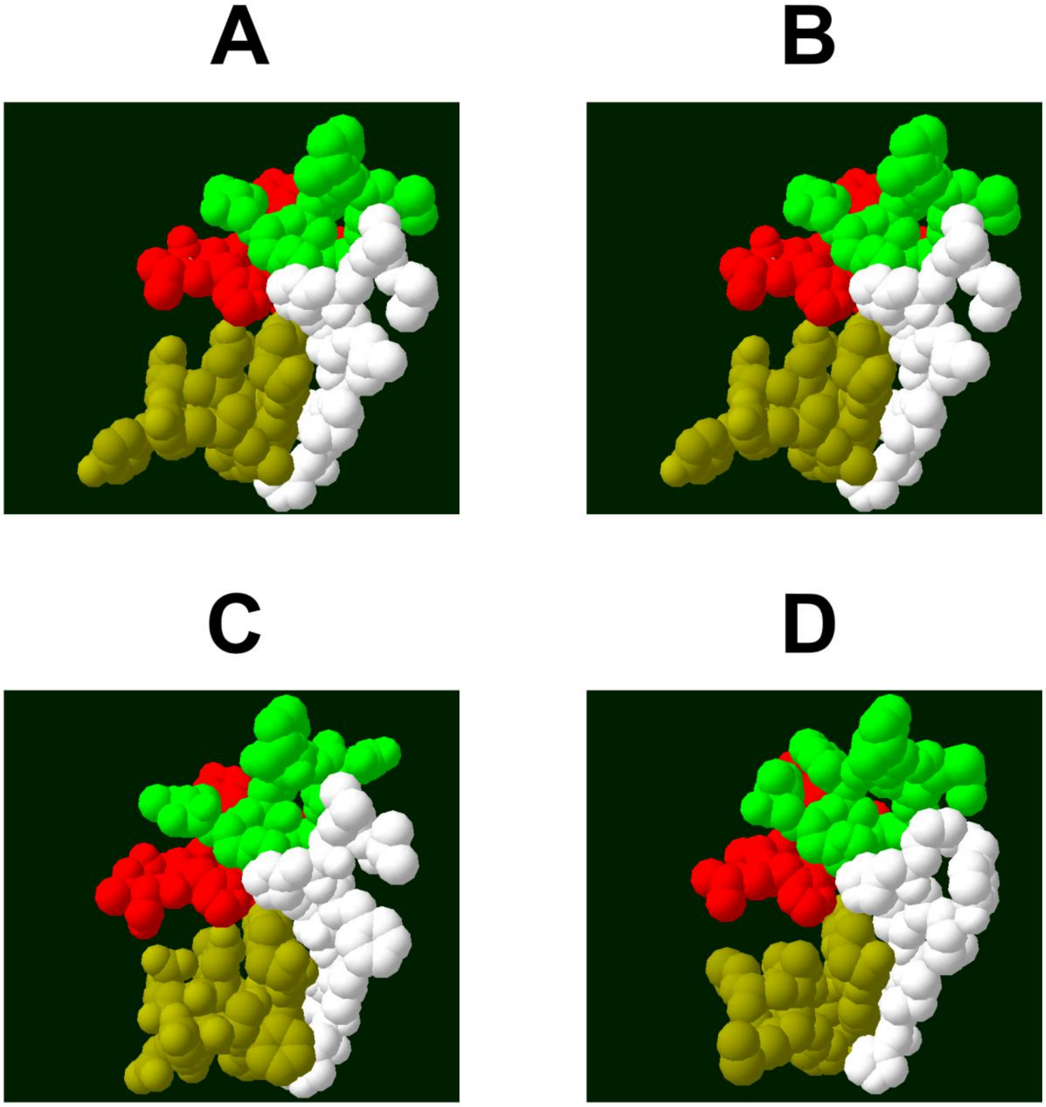

Supplementary Figure S14. The deduced structure of conserved domains in MCO proteins.

The conserved structure of domains to bind with Cu ion were predicted by using SWISS-MODEL Homology Modelling platform online (https://swissmodel.expasy.org/). The X-ray structure of spore cotA protein from B*acillus subtilis* (1gsk.1.A) was chosen to be the template to build MCO models. **A**. The domains of MCO from isolated strain ST7. **B**. Domains of cotA from *Bacillus*. The structure of three proteins were the same with each other, including the cotA of *Bacillus pumilus* strain WH4 (Su et al., 2013), cotA of *Bacillus subtilis*_strain 168 reported by Hullo (2001), and MCO of the reference *B. safensis* (WP_073204070.1). **C**. mofA from *Leptothrix discophora* strain SS-1 (Corstjens et al., 1997). **D**. moxA of *Pedomicrobium* sp. strain ACM3067 (Ridge et al., 2007).The other structure models of MCOs could not be predicted because the amino acid identities were too much diverse from that of strain ST7, such as mnxG, cumA, MCO-2447 and MCO-2556 so on.
